# Does chronic polyphenol supplementation modify resistance training-induced strength and muscle morphology adaptations in adults? A systematic review and multilevel meta-analysis of randomized controlled trials

**DOI:** 10.3389/fnut.2026.1906384

**Published:** 2026-08-28

**Authors:** Yang Zhu, Ning Shi, Chengbin Wang, Jiaming Yan, Hao Chen, Zheng Yi

**Affiliations:** 1 Institute of Physical Education and Training, Capital University of Physical Education and Sports, Beijing, China; 2 Tianjin Vocational College of Sports, Tianjin, China; 3 Shandong Provincial Shooting and Cycling Sports Management Center, Jinan, Shandong, China; 4 Xinjiang Sports Vocational and Technical College, Urumqi, Xinjiang, China; 5 School of Physical Education, Wuhan Business University, Wuhan, China

**Keywords:** exercise nutrition, lean mass, muscle hypertrophy, muscle strength, polyphenols, resistance training, three-level meta-analysis

## Abstract

**Background:**

Polyphenol-rich supplements are widely used to support exercise recovery, but whether these recovery-oriented effects translate into long-term resistance-training adaptations remains unclear.

**Objective:**

To determine whether chronic polyphenol supplementation modifies resistance-training-induced strength, muscle quantity/lean mass, and direct muscle morphology adaptations in adults.

**Methods:**

Following PRISMA guidelines, PubMed, Embase, Web of Science, the Cochrane Library, and Scopus were searched from inception to May 21, 2026. Randomized controlled trials in adults were included if they examined chronic oral polyphenol or polyphenol-rich supplementation combined with structured resistance training vs. placebo, a matched comparator, or resistance training alone. Risk of bias, methodological quality, and certainty of evidence were assessed using RoB 2, PEDro, and GRADE. Pooled effects were estimated in R 4.5.1 using three-level random-effects models with cluster-robust variance estimation.

**Results:**

Eleven reports describing 11 randomized trials and 11 independent study clusters, comprising 360 unique participants, were included. Strength adaptations showed a small, statistically uncertain estimate (22 effect sizes, 8 clusters, 257 unique participants; Hedges' g = 0.25, 95% CI −0.01 to 0.51, *P* = 0.060; 95% PI −0.36 to 0.86; *I*^2^within = 0.0%, *I*^2^between = 34.5%; low certainty). Muscle quantity/lean mass also showed a small, statistically uncertain estimate (13 effect sizes, 9 clusters, 280 unique participants; g = 0.13, 95% CI −0.07 to 0.33, *P* = 0.211; low certainty). Direct morphology was based on only three clusters and yielded an imprecise estimate close to zero (6 effect sizes, 115 unique participants; g = 0.05, 95% CI −0.21 to 0.30, *P* = 0.718; very low certainty). Exploratory subgroup analyses, sensitivity analyses, and publication-bias diagnostics did not materially change the primary interpretation.

**Conclusion:**

Current evidence does not establish a reliable additional effect of chronic polyphenol supplementation on resistance training-induced strength, lean-mass, or direct muscle-morphology adaptations. However, the limited number of independent trials, substantial clinical and methodological indirectness, and low-to-very-low certainty of evidence prevent firm conclusions regarding either benefit or lack of benefit.

## 1 Introduction

Resistance training represents the principal non-pharmacological strategy for increasing muscle strength, preserving lean mass, and attenuating age-related declines in neuromuscular function. Its adaptive effects are mediated by progressive mechanical loading, neural remodeling, muscle protein turnover, and, when the training stimulus is sustained, measurable changes in muscle size and architecture (1–3). These adaptations are relevant to both performance and health because muscle strength and muscle mass are linked to physical function, metabolic health, and age-related resilience (4, 5). Accordingly, whether non-protein bioactive compounds can modify resistance training-induced adaptations remains an unresolved question in exercise and nutrition science (6, 7).

Polyphenols are a structurally diverse class of plant-derived bioactive compounds, including flavonoids (flavonols, catechins, anthocyanins), isoflavones, curcuminoids, and related phenolic structures (8). Proposed mechanisms relevant to exercise include antioxidant activity, anti-inflammatory signaling, modulation of vascular function, mitochondrial remodeling, and attenuation of post-exercise oxidative and inflammatory stress (9). Acute recovery trials have reported mixed findings: some models demonstrated improved strength recovery following tart cherry or pomegranate supplementation (10–12), whereas randomized evidence is not uniformly supportive, with one trial in non-resistance-trained men observing no clear recovery benefit (13). Earlier reviews and meta-analyses of these short-term recovery studies have broadly concluded that polyphenol-rich foods or flavonoid-containing supplements may improve selected recovery outcomes after exercise-induced muscle damage, although effect sizes vary substantially by polyphenol source, dose, supplementation timing, exercise model, and outcome measurement (14, 15).

However, acute recovery from exercise-induced muscle damage should not be interpreted as equivalent to long-term training adaptation (16). Reactive oxygen and nitrogen species and transient inflammatory responses serve dual roles in skeletal muscle physiology (17): although excessive production can impair cellular integrity, controlled generation participates in redox-sensitive signaling, stress-response activation, mitochondrial remodeling, protein turnover, and skeletal muscle adaptation (18–20). Some antioxidant vitamin trials have reported altered training-related signaling or attenuated selected adaptations, including lean body mass responses in older adults (21), suggesting that the redox environment is a meaningful determinant of adaptation (22); however, these effects are not consistent across outcomes or populations. Polyphenols differ mechanistically from isolated antioxidant vitamins in that their actions may depend on indirect signaling pathways, gut-derived metabolites, vascular effects, formulation, and compound-specific bioavailability rather than direct free-radical scavenging (23, 24). Consequently, chronic polyphenol supplementation may support resistance training adaptation by limiting excessive physiological stress, or, conversely, may attenuate adaptive signaling depending on compound class, dose, supplementation timing, formulation, and population-specific factors such as training status and age (9, 25).

Previous systematic reviews have examined polyphenols in relation to exercise performance, acute recovery, exercise-induced muscle damage, sarcopenia, body composition, and broad exercise-induced physiological adaptations (26–28). Martinez-Negrin et al. synthesized exercise-training studies and found no significant overall effects of dietary polyphenols on muscle strength, lean mass gains, or peak power output, although exercise capacity was enhanced and compound-specific effects—particularly for isoflavones—were reported (9). Other reviews have focused on recovery after exercise-induced muscle damage, tart cherry and pomegranate supplementation, quercetin supplementation, polyphenol-rich berry products, functional phenolics, or sarcopenia-related outcomes (14, 15). Long-term trials combining resistance training with polyphenol supplementation have tested tea-derived polyphenols, quercetin glycosides, matcha, curcumin, epicatechin, currant-derived polyphenols, green tea, and soy isoflavones across heterogeneous adult populations, with inconsistent findings for strength, muscle mass, cross-sectional area, fatigue, stiffness, mitochondrial outcomes, and body composition (29–34). Although previous reviews have included strength- and lean-mass-related outcomes within broader exercise contexts, the evidence specific to chronic polyphenol supplementation during resistance training has not been comprehensively synthesized using an updated, resistance-training-focused framework.

A further limitation of the current evidence base is statistical dependence arising from trials that contribute multiple effect sizes across strength tests, anatomical sites, dose arms, or overlapping participant samples—a structural feature that conventional two-level meta-analyses do not adequately accommodate. The present systematic review and three-level meta-analysis therefore aimed to determine whether chronic polyphenol supplementation modifies resistance training-induced adaptations in strength, muscle mass or lean mass, and direct muscle morphology in adults. If beneficial effects exist, they were hypothesized to be more detectable for strength-related than for morphological outcomes, as neural and functional adaptations may respond to a broader range of physiological stimuli than structural hypertrophy, and because prior short-term evidence most consistently supports polyphenol effects on muscle function rather than muscle size.

## 2 Materials and methods

### 2.1 Registration

This systematic review and meta-analysis was conducted in accordance with the PRISMA 2020 statement and the methodological recommendations of the Cochrane Handbook for Systematic Reviews of Interventions (35). The protocol was prospectively registered in PROSPERO (CRD420261334989).

### 2.2 Search strategy

A systematic search was conducted in PubMed, Embase, Web of Science, the Cochrane Library, Scopus from database inception to May 21, 2026. Search terms were structured around three core concepts: polyphenol exposure, resistance training, and randomized or controlled trial design. Polyphenol-related terms included polyphenol, flavonoid, phenol, anthocyanin, catechin, resveratrol, quercetin, curcumin, isoflavone, and common polyphenol-rich foods or extracts. Training-related terms included resistance training, resistance exercise, strength training, weight training, progressive resistance training, and related resistance-exercise modalities. Trial-design terms included randomized, placebo, trial, crossover, and controlled trial. Outcome terms and supplementation-duration restrictions were not applied at the search stage to maximize sensitivity. Reference lists of eligible articles and relevant reviews were screened manually. Full search strategies and database-specific yields are provided in Supplementary Table S1.

### 2.3 Eligibility criteria

Explicit inclusion and exclusion criteria were established a priori according to the PICOS framework (Population, Intervention, Comparator, Outcomes, and Study design) for systematic reviews (36).

#### 2.3.1 Inclusion criteria

(1) Participants were adults aged ≥18 years. Eligible populations included healthy adults, athletes, middle-aged or older adults, and adults with sarcopenia or reduced muscle function.

(2) The intervention consisted of chronic oral supplementation with polyphenols or polyphenol-rich foods or extracts administered concurrently with structured resistance training for ≥4 weeks.

(3) Resistance training was the principal adaptive stimulus, and comparator groups received identical or closely matched training. Strict-primary analyses included only comparisons that isolated the incremental effect of polyphenol supplementation. Comparisons against an active nutritional background were retained only for extended analyses when the incremental polyphenol effect remained identifiable.

(4) Studies reported at least one extractable long-term outcome related to neuromuscular performance, muscle quantity, direct muscle morphology, or body composition. Grip strength, power, jumping performance, and functional outcomes were eligible for secondary synthesis but were excluded from the primary strength domain.

(5) Eligible designs were randomized controlled trials.

#### 2.3.2 Exclusion criteria

(1) Studies were excluded if adult data could not be extracted separately from pediatric, adolescent, animal, or *in vitro* data.

(2) Studies were excluded if endurance training, high-intensity interval training, concurrent training, rehabilitation, or multicomponent lifestyle interventions predominated and the resistance-training effect could not be isolated.

(3) Studies evaluating only acute supplementation, single-exercise responses, or short-term recovery outcomes were excluded.

(4) Studies using multicomponent supplements were excluded when the independent polyphenol effect could not be separated from other active ingredients.

(5) Non-randomized studies, observational studies, reviews, case reports, non-original reports, duplicate publications without independent data, and reports without extractable outcomes were excluded.

### 2.4 Study selection

All records retrieved from electronic databases were imported into EndNote X9 for deduplication. After duplicate removal, two reviewers (NS, CBW) independently screened titles and abstracts against the predefined eligibility criteria. Full texts were obtained for all potentially eligible reports and independently assessed by the same reviewers. Disagreements were resolved through discussion; unresolved disagreements were adjudicated by a third reviewer (JMY). Inter-reviewer agreement at the full-text stage was quantified using Cohen's κ (37). The study-selection process and reasons for full-text exclusion were documented using a PRISMA 2020 flow diagram.

### 2.5 Data extraction

#### 2.5.1 Data extraction procedures

Two reviewers (NS, CBW) independently extracted data using a standardized Microsoft Excel form and cross-checked all entries before analysis. Extracted information included study and participant characteristics, sample size, sex distribution, age, body mass index, resistance-training protocol, polyphenol formulation and dose, intervention duration, comparator condition, co-interventions, outcome definitions, and the numerical data required for effect-size calculation. Disagreements were resolved by discussion and, when necessary, adjudicated by a third reviewer (JMY). For continuous outcomes, group-specific means, standard deviations, and sample sizes were extracted at baseline and post-intervention. Author-reported change scores and change-score standard deviations were prioritized. Numerical values reported only in figures were extracted using WebPlotDigitizer and independently verified by two reviewers (38). Shared-control multi-arm trials, reports derived from the same or overlapping samples, co-supplementation designs, figure-estimated data, and mixed resistance training plus HIIT protocols were flagged before analysis. Reports derived from the same participant source were assigned to a common study cluster to prevent double-counting (39).

#### 2.5.2 Outcome domain classification and handling

Outcome domains were pre-specified during PROSPERO registration and operationally classified before effect-size calculation. Strength included dynamic repetition-maximum, isometric/dynamometer, and isokinetic measures. Muscle quantity/lean mass comprised whole-body, appendicular, or regional lean- or muscle-mass estimates derived from body-composition assessments, whereas CSA/direct morphology comprised localized muscle thickness, whole-muscle cross-sectional area, and muscle-fiber cross-sectional area. These domains were analyzed separately and were not treated as interchangeable. Measurement modality was coded separately. Distinct eligible outcomes were retained, duplicate representations were removed, and statistical dependence was modeled at the study-cluster level (40).

### 2.6 Quality assessment and risk of bias assessment

Two reviewers independently assessed risk of bias using the revised Cochrane risk-of-bias tool for randomized trials (RoB 2) (41). The five domains were the randomization process, deviations from intended interventions, missing outcome data, outcome measurement, and selection of the reported result. Overall judgments were classified as low risk, some concerns, or high risk. Methodological quality was additionally summarized using the PEDro scale (42). Items 2–11 contributed to the total score, with scores ≥6, 4–5, and ≤ 3 interpreted as high, moderate, and low quality, respectively. PEDro scores were used descriptively and did not replace domain-based RoB 2 judgments.

### 2.7 GRADE evidence assessment

The certainty of evidence for the three primary outcome domains was assessed using GRADE (43). Evidence from randomized trials initially received a high-certainty rating and was downgraded, when appropriate, for risk of bias, inconsistency, indirectness, imprecision, and publication bias. Judgments incorporated RoB 2 findings, pooled estimates, confidence intervals, heterogeneity components, prediction intervals, sensitivity analyses, and exploratory small-study-effect diagnostics.

### 2.8 Statistical analysis

All analyses were conducted in R version 4.5.1 (R Foundation for Statistical Computing, Vienna, Austria) using the “metafor,” “clubSandwich,” and “ggplot2” packages (44). The primary effect measure was Hedges' g, calculated as the small-sample-corrected standardized between-group difference in pre-to-post change scores (45). Author-reported change scores and change-score standard deviations (SDs) were prioritized over post-intervention data. Mean change was defined as Δ*M* = *M*_*post*_−*M*_*pre*_. When the change-score SD was unavailable, it was estimated as (45, 46):

$$\begin{array}{l} SD_{\Delta }=\sqrt{SD_{pre}^{2}+SD_{post}^{2}-2rSD_{pre}SD_{post}} \end{array}$$

using *r* = 0.5 in the primary analysis (47). Hedges' g was calculated as:

$$\begin{array}{l} g_{\Delta }=J\left(df\right)\frac{\Delta M_{EXP}-\Delta M_{CON}}{\sqrt{\left[\left(n_{EXP}-1\right)SD_{\Delta ,EXP}^{2}+\left(n_{CON}-1\right)SD_{\Delta ,CON}^{2}\right]/df}} \end{array}$$

Where *df* = *n*_*EXP*_+*n*_*CON*_−2 and *J*(*df*) is the exact small-sample correction factor (48). Change-score standardization was retained to align with the extracted change data; sensitivity to the assumed correlation and standardizer was examined in alternative analyses. When change means and SDs were supplied as group-level inputs, escalc (measure = “SMD,” correct = TRUE) reproduced this point estimator, while vtype = “LS” specified its large-sample sampling-variance approximation. Absolute effect sizes were described as trivial (< 0.20), small (0.20–0.49), moderate (0.50–0.79), or large (≥0.80) (49).

When eligible intervention arms shared a common control group and represented the same polyphenol class and training context, intervention arms were combined in accordance with Cochrane guidance to avoid double-counting the control group. Distinct comparisons were retained when they involved independent participant groups or substantively different training conditions. Because several trials contributed multiple outcomes, anatomical sites, comparisons, dose arms, or reports derived from the same participant source, pooled effects were estimated using three-level random-effects models with restricted maximum likelihood estimation (50, 51).

$$\begin{array}{l} y_{ij}=\beta_{0}+u_{\left(3\right)j}+u_{\left(2\right)ij}+e_{ij} \end{array}$$

Level 1 represented sampling variance, level 2 within-cluster heterogeneity among dependent effects, and level 3 between-cluster heterogeneity. Pooled estimates were reported as Hedges' g, 95% confidence intervals, two-sided *P*-values, 95% prediction intervals, variance components, and decomposed *I*^2^ (50). Prediction intervals were calculated as: $\hat{\mu }\pm 1.96\sqrt{SE{\left(\hat{\mu }\right)}^{2}+\hat{\tau_{level2}^{2}}+\hat{\tau_{level3}^{2}}}$. using a standard-normal critical value without an additional degrees-of-freedom or heterogeneity-estimation uncertainty term. Coincident confidence and prediction intervals when both variance components were estimated as zero were not interpreted as evidence of homogeneous true effects. N denotes unique participants within each domain.

Because exact within-study sampling covariances were unavailable, the primary sampling-variance matrix was specified as *V* = *diag*(*v*_*i*_). Cluster-robust variance estimation with CR2 correction and Satterthwaite degrees of freedom was applied as a robustness check (52, 53), with cluster counts and effective degrees of freedom reported in Supplementary Table S8. Separate sensitivity models used block-diagonal covariance matrices assuming within-cluster sampling-error correlations of ρ = 0.30, 0.60, *and*0.90, with zero covariance between clusters. Effects were also aggregated to one estimate per cluster at ρ = 0.60 and analyzed using random-effects REML with Knapp–Hartung inference (54). CRVE findings for CSA/direct morphology were descriptive, and no moderator or meta-regression analysis was conducted for this three-cluster domain.

Sensitivity analyses included complete study-cluster deletion (55), targeted deletion of pre-specified clusters, data-feature exclusions, recalculation of missing change-score SDs using *r* = 0.30, 0.50, *and*0.70, and Morris's pooled baseline-SD estimator *d*_*ppc*2_ for outcomes with available baseline data (56). Publication bias and small-study effects were explored using multilevel Egger regression, with the effect-size standard error entered as a moderator. Traditional Egger tests, contour-enhanced funnel plots (57), and trim-and-fill analyses were additionally performed after aggregation to one domain-specific effect per study cluster (58). Because fewer than 10 clusters contributed to each primary outcome domain, publication-bias diagnostics were interpreted as exploratory (59). All tests were two-sided, with *P* < 0.05 indicating statistical significance.

## 3 Results

### 3.1 Study selection results

**Figure 1:**
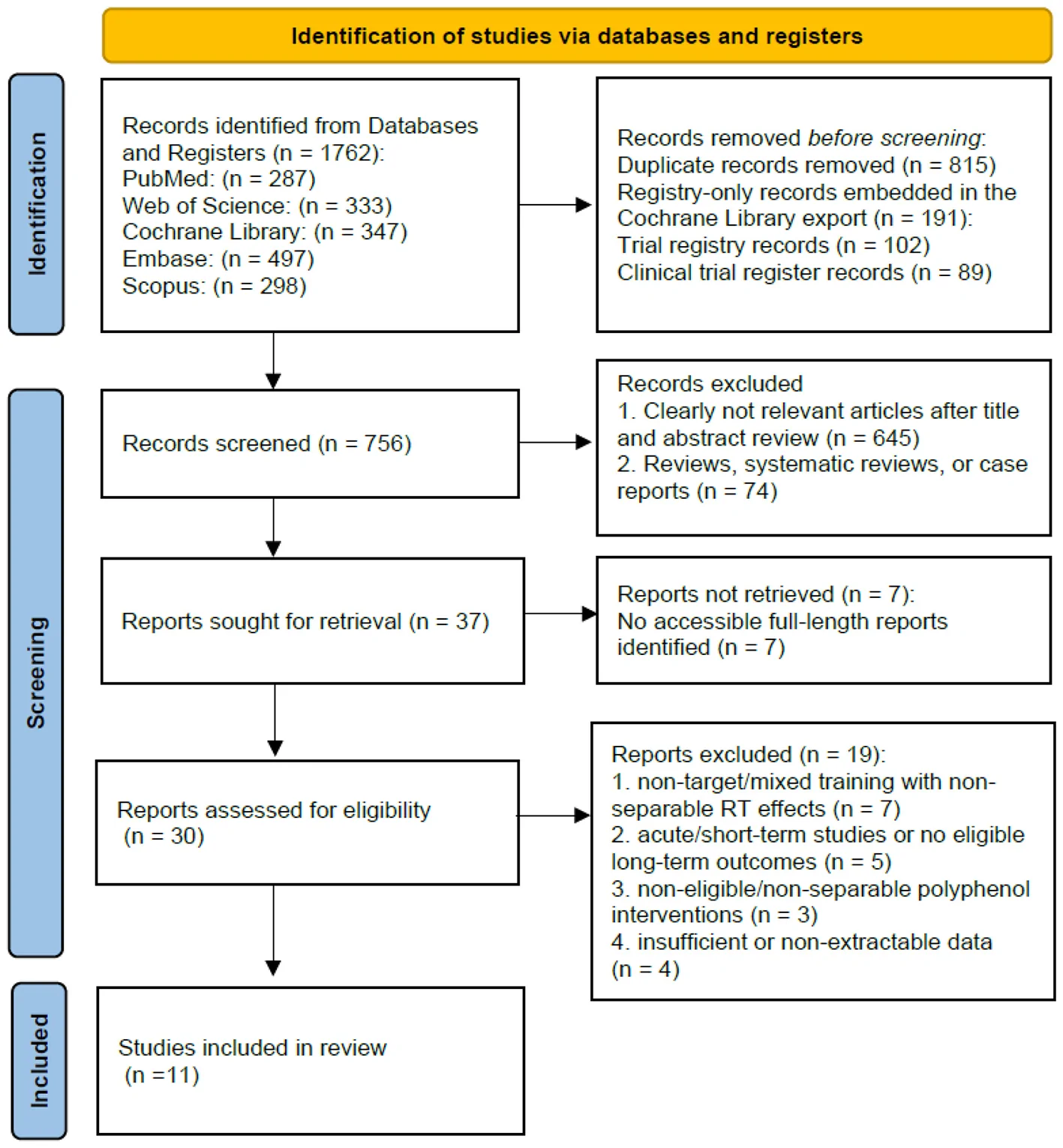
PRISMA flow diagram of study selection.

The search identified 1,762 records. After removing duplicates (*n* = 815), trial-registry records (*n* = 102), and clinical-trial records not constituting eligible reports (*n* = 89), 756 records were screened by title and abstract. Inter-reviewer agreement was almost perfect at both the title/abstract screening stage (Cohen's κ = 0.956) and the full-text screening stage (Cohen's κ = 0.850). After title/abstract screening, 37 reports were sought for retrieval, seven were unavailable, and 30 full texts were assessed. Nineteen reports were excluded because of non-separable training effects (*n* = 7, see Supplementary material), ineligible outcome timing (*n* = 5), ineligible or non-separable polyphenol interventions (*n* = 3), or insufficient data (*n* = 4). Eleven reports were included in the systematic review and meta-analysis (Figure 1).

### 3.2 Characteristics of included studies

This review included 11 reports describing 11 randomized trials and 11 analyzable study clusters, comprising 360 unique participants. Analytic sample sizes ranged from 17 participants in Shigeta et al. (34) Trial 1 to 51 participants in Tokuda and Mori (60). Sex was reported with extractable counts for 311 participants: 154 men and 157 women. Sex distribution for the four training arms extracted from the Juesas/Saez-Berlanga (32, 61) curcumin trial was not reported separately and was therefore classified as unknown for 49 participants. Trials were conducted in Denmark, Japan, Spain, Iran, the United States, and Brazil, and included untrained young men, healthy or sedentary older adults, sarcopenic older men, overweight or obese women, and postmenopausal women. Intervention duration ranged from 6 to 39 weeks.

Most studies used resistance training two or three times per week. Machine-based progressive resistance training was used by Beyer et al. (29), Cardoso et al. (31), Mafi et al. (62), Otsuka et al. (30), Shigeta et al. (34), and Orsatti et al. (63); Nishikawa et al. (64) used unilateral isometric knee-extension training; Juesas/Saez-Berlanga used high-resistance elastic-band training (32, 61); Tokuda and Mori used body-weight and elastic-band training (60); and Flensted-Jensen et al. combined resistance-based training with a limited HIIT component (33). Polyphenol exposures included red/blackcurrant polyphenols, quercetin glycosides, curcumin, matcha, tea catechins, epicatechin, green/black tea polyphenol blends, green-tea powder, and soy isoflavones. Training and supplement adherence, background diet, and implementation of progressive loading were inconsistently reported, limiting quantitative comparison and interpretation. Reported co-exposures included caffeine, essential amino acids, a hypocaloric dietary run-in, and limited HIIT. Safety data were available in five reports and systematically assessed only by Otsuka et al.; therefore, intervention safety could not be reliably evaluated (Supplementary Table S13). Study characteristics, interventions, and cluster mapping are presented in Tables 1, 2 and Supplementary Table S12. Study-level information on supplementation compliance, training attendance, withdrawals, dietary assessment, bioactive co-exposures, nutritional co-interventions, and implementation of load progression is summarized in Supplementary Table S14.

**Table 1:** Basic characteristics of the included studies.

Included studies	Country of origin	Research participant	Age (years)	Sample size		BMI (kg/m^**2**^)		Duration
				EXP	CON	EXP	CON	
Flensted-Jensen et al. (33)	Denmark	Healthy older adults	61 ± 4	10M/10F	11M/11F	26.2 ± 2.1	26.1 ± 3.3	12 weeks
Nishikawa et al. (64)	Japan	Healthy older adults	65–82	6M/7F	5M/8F	22.78 ± 4.66	21.51 ± 2.93	6 weeks
Juesas et al. (61) and Saez-Berlanga et al. (32)	Spain	Sedentary or minimally active older adults	≥60	10	10	26.2 ± 3.1	26.8 ± 2.9	16 weeks
				16	13	26.3 ± 3.4	25.7 ± 3.4	
Shigeta et al. (34)	Japan	Healthy untrained young men	22.3 ± 0.3	9	8	20.4 ± 2.0	19.8 ± 2.6	8 weeks
			20.9 ± 1.3	9	10	19.7 ± 1.2	21.6 ± 2.1	12 weeks
Otsuka et al. (30)	Japan	Community-dwelling middle-aged and older adults	50–74	7M/9F	8M/8F	22.56 ± 5.98	22.08 ± 3.61	24 weeks
				7M/9F		22.56 ± 5.98		
Tokuda and Mori (60)	Japan	Healthy older adults	≥65	3M/23F	3M/22F	22.1 ± 2.6	22.4 ± 3.2	24 weeks
Mafi et al. (62)	Iran	Sarcopenic older men	65–75	15M	14M	24.05 ± 2.55	23.20 ± 1.94	8 weeks
Beyer et al. (29)	USA	Men without systematic RT in the previous year	21.9 ± 2.5	14M	15M	23.70 ± 2.92	27.05 ± 5.33	6 weeks
Cardoso et al. (31)	Brazil	Overweight or obese women	20–40	9F	9F	30.4 ± 1.9	31.8 ± 1.9	8 weeks
Orsatti et al. (63)	Brazil	Sedentary postmenopausal women	45–70	15F	18F	30.3 ± 4.7	26.0 ± 3.0	39 weeks

M, Male; F, Female; EXP, Experimental group; CON, Control group.

**Table 2:** Summary of interventions and outcome measures of the included studies.

Included studies	Resistance-training intervention	Polyphenol supplementation	Comparator/ placebo	Outcomes used in this review
Flensted-Jensen et al. (33)	12 wk supervised training, 3 sessions/wk: two whole-body RT sessions and one lower-body RT session followed by brief cycle HIIT; RT progressed from 15RM to heavier 6–10RM loading.	Red-/blackcurrant polyphenol drink, approximately 700 mg total polyphenols/d; 30-d loading before training, then continued daily during training.	Taste-, color-, macronutrient-, and container-matched liquid placebo without polyphenols.	MVC; whole-body and thigh lean mass; VL CSA; type I and type II fiber CSA; fat mass.
Nishikawa et al. (64)	6 wk unilateral isometric knee-extension RT, 3 sessions/wk; each leg performed 3 × 10 contractions at 60% baseline MVF with real-time force feedback.	Quercetin glycosides, 200 mg/d, as two-colored gelatin capsules taken every morning.	Visually and olfactorily matched colored capsules containing dextrin.	VL/VI muscle thickness; right lower-limb muscle mass/volume.
Juesas et al. (61) and Saez-Berlanga et al. (32)	16 wk high-resistance elastic-band RT, 3 sessions/wk. Aecc used low/unloaded concentric action plus 5-s accentuated eccentric action; Max used 2-s concentric and 2-s eccentric actions at RPE 7–8, approximately 85% 1RM.	Curcumin formulation (Cursol), 500 mg/d as two 250-mg capsules after breakfast and dinner; equivalent to 10.5 mg/d curcumin.	Identical capsules containing maltodextrin and excipients.	Whole-body muscle mass and fat mass; elbow flexion and extension peak torque at 60 deg/s.
Shigeta et al. (34)	Trial 1: 8 wk; Trial 2: 12 wk. Machine-based RT, 2 sessions/wk; eight exercises, 3 sets at individual 10RM with progressive adjustment.	Matcha beverage twice daily; each drink contained 1.5 g matcha powder. Daily intake: 3.0 g matcha, approximately 333 mg total polyphenols, 189 mg EGCG, and 90 mg caffeine.	Tea-flavored placebo beverage matched for carbohydrate and caffeine, without matcha powder.	Trial 1: leg press 10RM, chest press 10RM, knee-extension strength, skeletal muscle mass. Trial 2: skeletal muscle mass and body composition.
Otsuka et al. (30)	24 wk low-intensity machine RT, 3 sessions/wk; leg extension, leg curl, leg press, and chest press at 40% 1RM, 3 × 14 repetitions.	Quercetin glycosides, 200 mg/d or 500 mg/d, taken as six same-color capsules; two active dose arms shared one placebo group.	Six same-color placebo capsules containing 0 mg QG; capsule mass/volume matched using dextrin, silicon oxide, and calcium stearate.	Chest press, leg press, leg extension, and leg curl 1RM; thigh CSA; VL CSA; whole-body and regional lean mass.
Tokuda and Mori (60)	24 wk body-weight and elastic-band RT, 2 sessions/wk; 20-min warm-up plus 40-min RT; load progressed from approximately 50% to 70% 1RM.	Tea-catechin add-on: 540 mg catechins after each exercise session, ingested within 30 min post-exercise; all active-arm participants also received 3,000 mg EAA.	No placebo; additive comparison was Ex + EAA + tea catechins vs. Ex + EAA.	Knee-extension strength
Mafi et al. (62)	8 wk supervised machine-based RT, 3 sessions/wk; eight upper- and lower-body exercises, 3 × 8–12 repetitions at 60–80% 1RM.	Epicatechin, 1 mg/kg/d, once daily for 8 wk.	Main comparison was RT + epicatechin vs. RT alone; the separate non-training placebo arm used matched starch capsules.	Leg press 1RM; chest press 1RM.
Beyer et al. (29)	6 wk full-body progressive RT, 3 sessions/wk, after a 4 wk supplementation loading phase; core lifts at approximately 70% 1RM and accessory exercises at 8–10RM.	Green/black tea polyphenol blend, 2,000 mg/d in two doses; at least 40% total polyphenols, with EGCG, theaflavins, caffeine, and manganese reported.	Identical gelatin capsules containing microcrystalline cellulose.	Squat, leg press, and leg extension 1RM, reported relative to body mass.
Cardoso et al. (31)	8 wk RT, 3 sessions/wk, after a 4 wk hypocaloric diet run-in; 10 exercises, 3 × 10 repetitions, approximately 70% 1RM.	Green-tea powder, 20 g/d in two 10-g servings; daily intake approximately 320 mg total polyphenols and 40 mg caffeine.	Powdered placebo matched for nutrition, enrichment, color, odor, texture, and taste, but without green-tea powder, polyphenols, or caffeine.	Lean body mass; body fat; 45-degree leg press, bench press, and back pull-down strength.
Orsatti et al. (63)	39 wk supervised whole-body RT, at least 2 sessions/wk; 3 × 8–12 repetitions at 60–80% 1RM after familiarization, with progressive load adjustment.	Soy isoflavone extract, 100 mg/d isoflavones in glycoside form, provided as two capsules/d; approximately 50% genistein and 35% daidzein.	Two lactose capsules/d, identical in appearance to active capsules.	Leg extension 1RM; whole-body and limb muscle mass; whole-body and trunk fat mass; body fat percentage.

Aecc, accentuated eccentric training; AppMMI, appendicular skeletal muscle mass index; CSA, cross-sectional area; EAA, essential amino acids; EGCG, epigallocatechin gallate; HIIT, high-intensity interval training; Max, maximal strength training; MVC, maximal voluntary contraction; MVF, maximal voluntary force; QG, quercetin glycoside; QOL, quality of life; RM, repetition maximum; RPE, rating of perceived exertion; RT, resistance training; SMI, skeletal muscle mass index; TUG, timed up-and-go test; VI, vastus intermedius; VL, vastus lateralis. Tokuda and Mori was retained as an add-on nutrition comparison and sensitivity/secondary-design case; it was not treated as a pure polyphenol-placebo contrast in the strict primary interpretation.

### 3.3 Risk of bias and quality assessment results

**Figure 2:**
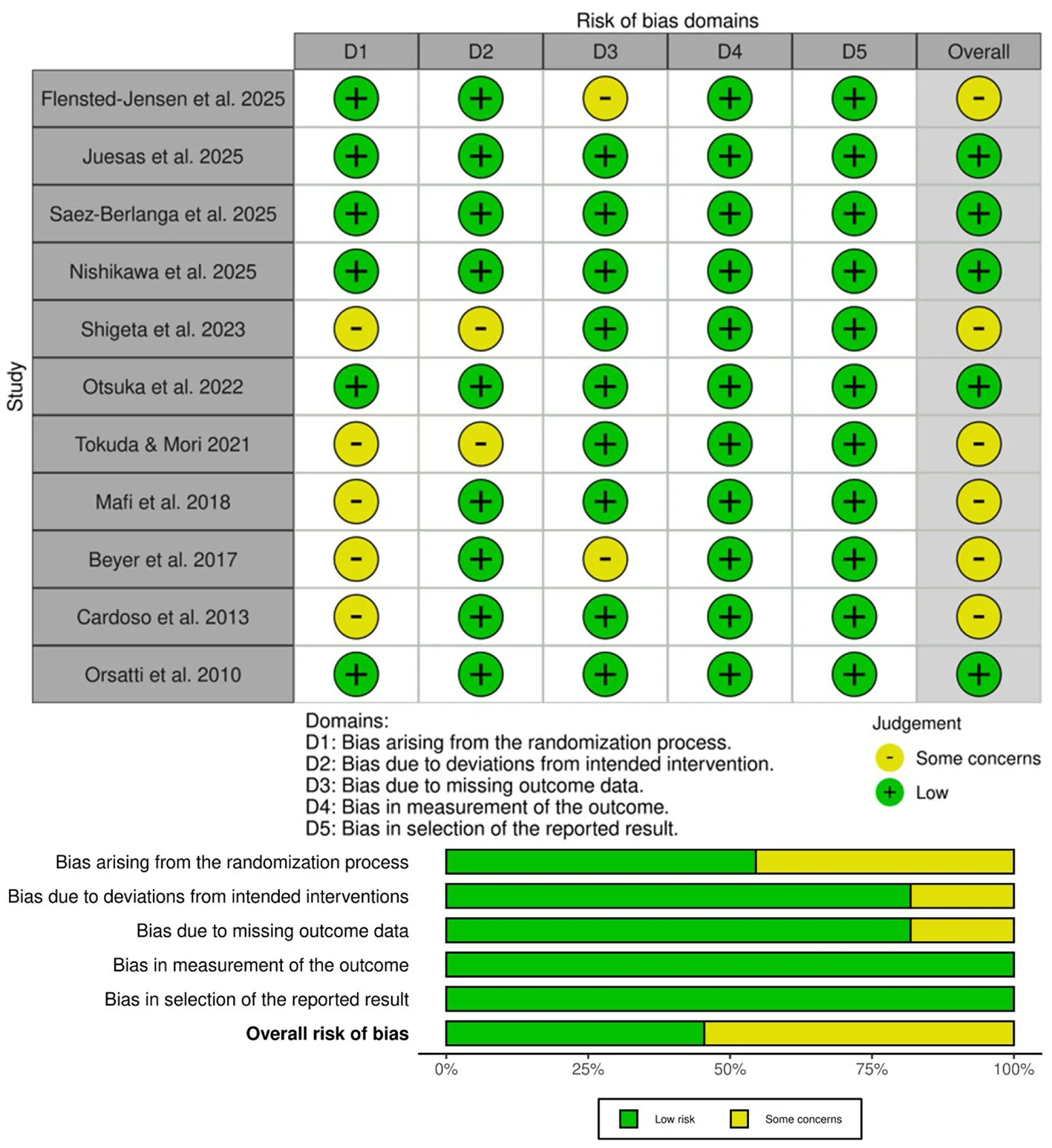
Risk of bias assessment of the included studies.

Overall risk of bias was low in five reports (45.5%) and raised some concerns in six (54.5%); no report was rated at high risk (Figure 2). Agreement between reviewers was substantial to almost perfect for RoB 2 domains (κ = 0.68–1.00). Concerns primarily involved incomplete reporting of randomization or allocation procedures, deviations from intended interventions, and missing outcome data. Random allocation in Cardoso et al. (31) was confirmed from the associated Master's dissertation (65), although sequence generation and allocation concealment remained insufficiently described. All reports were rated at low risk for outcome measurement and selection of reported results. PEDro scores ranged from 6 to 10 (median, 8; κ = 0.90), with deductions mainly related to allocation concealment, blinding, and intention-to-treat analysis (Table 3). PEDro was used only as a supplementary assessment of methodological features and was not interpreted as a risk-of-bias instrument or as overriding RoB 2 judgements. Overall, the evidence base showed generally adequate methodological conduct but retained study-specific concerns relevant to interpretation.

**Table 3:** Methodological quality assessment of the included studies based on the PEDro scale.

Study	Eligibility^*^	2	3	4	5	6	7	8	9	10	11	Total (/10)
Flensted-Jensen et al. (33)	1	1	1	1	1	1	1	0	0	1	1	8
Juesas et al. (61)	1	1	1	1	1	1	1	1	0	1	1	9
Saez-Berlanga et al. (32)	1	1	1	1	1	1	1	1	1	1	1	10
Nishikawa et al. (64)	1	1	1	1	1	1	1	1	0	1	1	9
Shigeta et al. (34)	1	1	0	1	0	0	0	1	1	1	1	6
Otsuka et al. (30)	1	1	1	1	1	1	1	1	0	1	1	9
Tokuda and Mori (60)	1	1	0	1	0	0	1	1	0	1	1	6
Mafi et al. (62)	1	1	0	1	1	0	0	1	1	1	1	7
Beyer et al. (29)	1	1	0	0	1	1	1	0	0	1	1	6
Cardoso et al. (31)	1	1	0	1	1	1	1	1	1	1	1	9
Orsatti et al. (63)	1	1	1	1	1	0	1	1	0	1	1	8

^*^Eligibility is reported separately and is not included in the total PEDro score. Items 2–11 denote random allocation, concealed allocation, baseline comparability, participant blinding, therapist blinding, assessor blinding, adequate follow-up, intention-to-treat analysis, between-group comparisons, and reporting of point estimates and variability, respectively. A value of 1 indicates that the criterion was satisfied; 0 indicates that it was not satisfied. PEDro scores were treated as supplementary methodological descriptors and not as risk-of-bias classifications.

### 3.4 Three-level meta-analysis results

#### 3.4.1 Primary outcome effects

**Figure 3:**
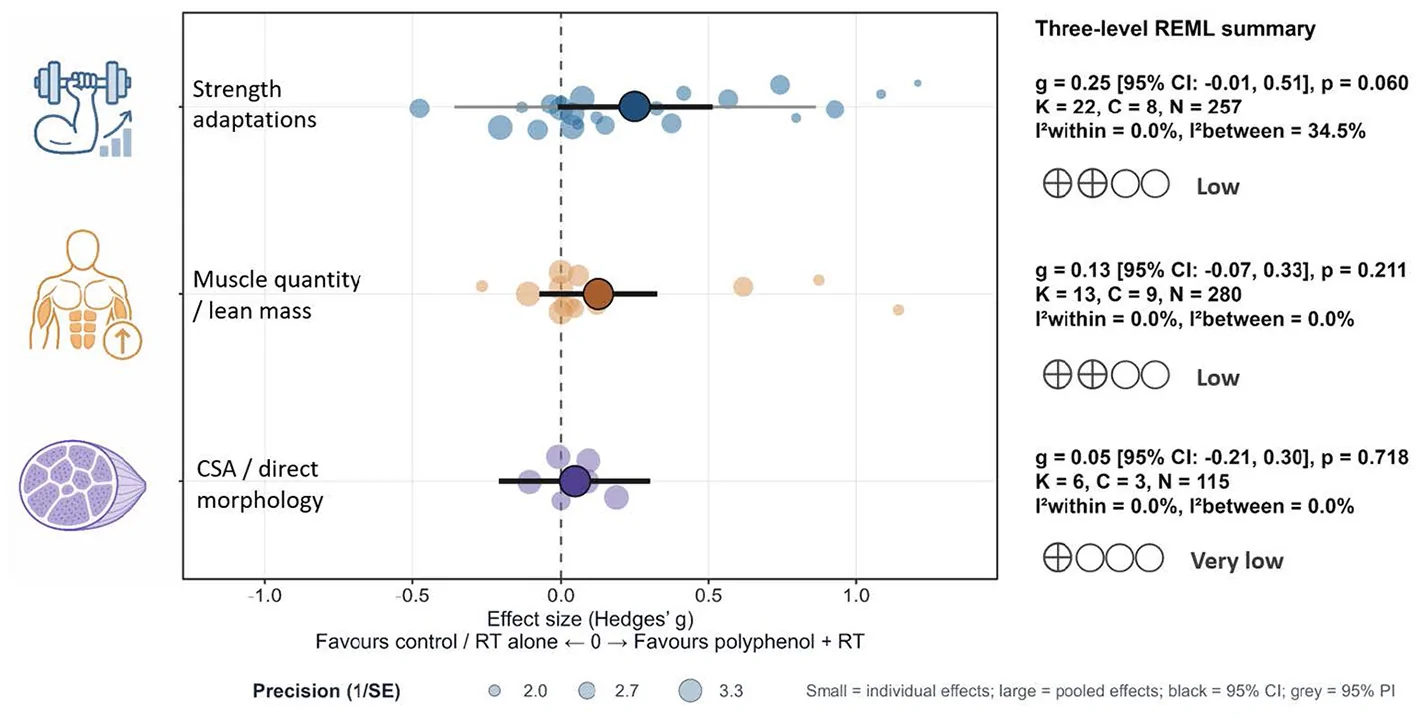
Orchard plot of three-level meta-analytic effects of chronic polyphenol supplementation on resistance-training-induced strength, muscle quantity, and muscle morphology adaptations.

**Figure 4:**
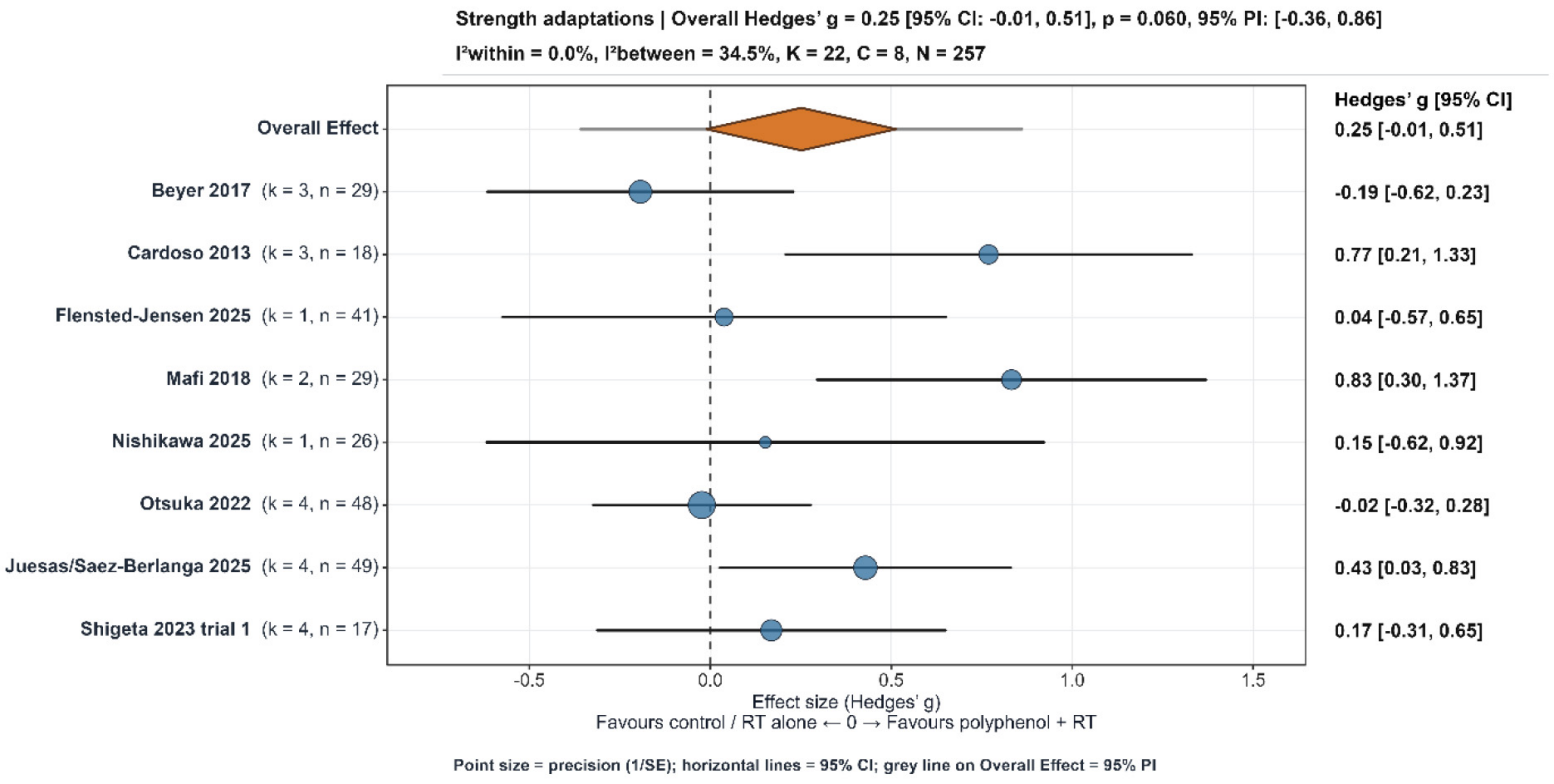
Study-cluster forest plot for the effect of chronic polyphenol supplementation on resistance-training-induced strength adaptations.

**Figure 5:**
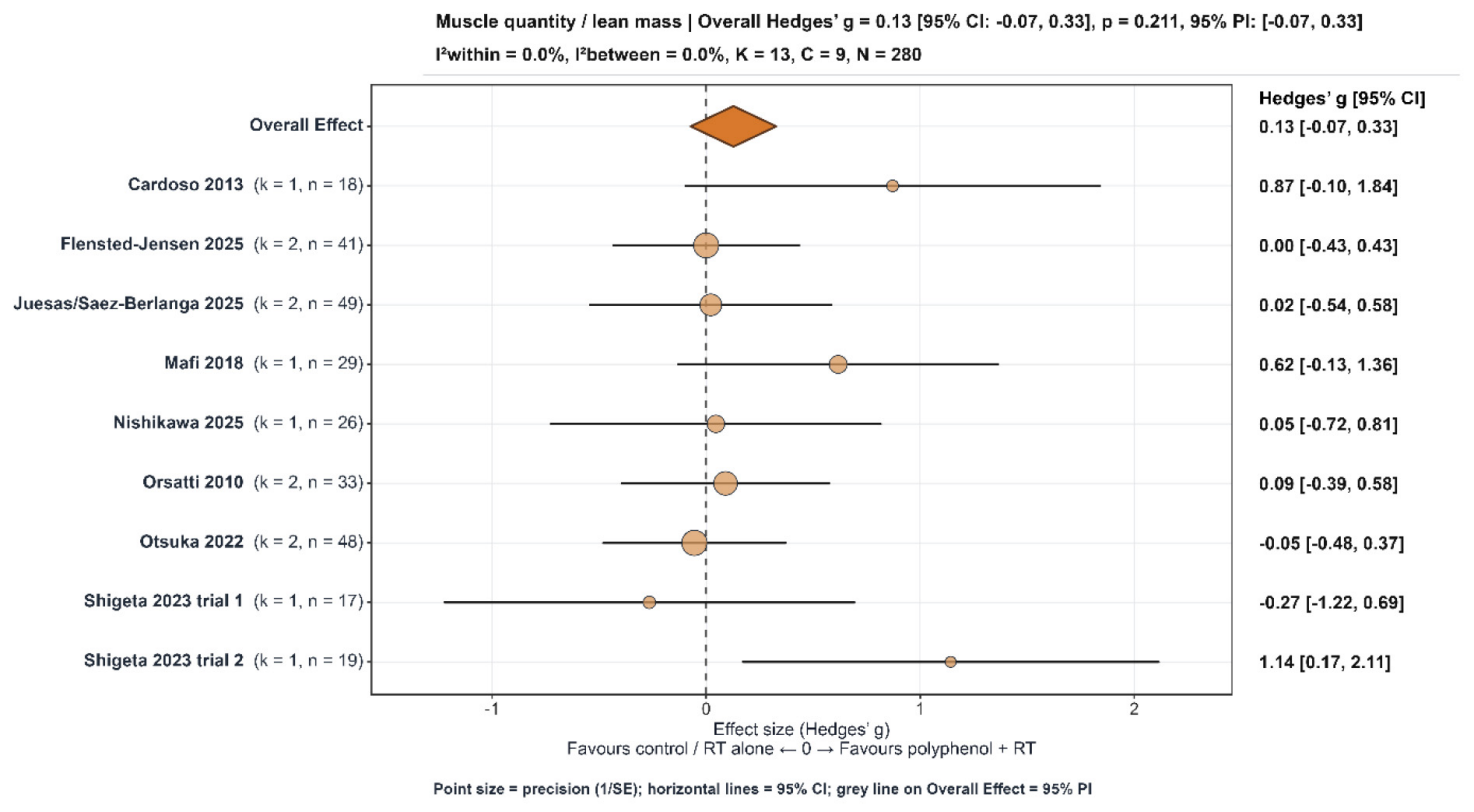
Study-cluster forest plot for the effect of chronic polyphenol supplementation on muscle quantity and lean mass adaptations.

**Figure 6:**
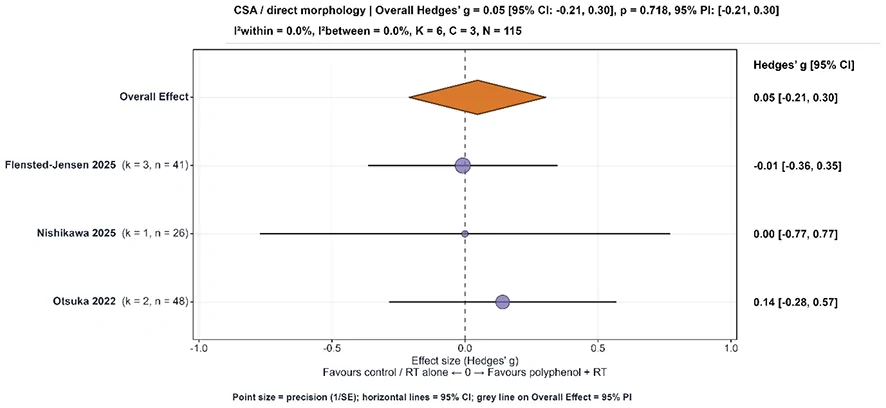
Study-cluster forest plot for the effect of chronic polyphenol supplementation on CSA and direct muscle morphology adaptations.

Three separate three-level random-effects models were fitted for the primary outcome domains, with positive values indicating more favorable adaptations in the polyphenol plus resistance-training group. Muscle quantity/lean mass represented body-composition-derived estimates, whereas CSA/direct morphology represented localized assessments of muscle structure. The pooled estimates are summarized in the orchard plot (Figure 3), and the study-cluster-level forest plots are shown separately for strength adaptations, muscle quantity/lean mass, and CSA/direct morphology (Figures 4–6). Detailed effect-size-level forest plots are provided in Supplementary Figures S1–S3.

For strength adaptations, 22 effect sizes from 8 study clusters and 257 participants yielded a small (29–34, 61, 62, 64), directionally positive but statistically uncertain effect (Hedges' g = 0.25, 95% CI −0.01 to 0.51, *p* = 0.060). The 95% prediction interval was wide (−0.36 to 0.86), and the CRVE robustness check was non-significant (*p* = 0.105). Heterogeneity was mainly located between study clusters (*I*^2^within = 0.0%, *I*^2^between = 34.5%). For muscle quantity/lean mass, 13 effect sizes from 9 study clusters and 280 participants showed a small non-significant effect (g = 0.13, 95% CI −0.07 to 0.33, *p* = 0.211) (30–34, 61–64). The 95% prediction interval was narrow and crossed the null (−0.07 to 0.33), with negligible heterogeneity (*I*^2^within = 0.0%, *I*^2^between = 0.0%). For CSA/direct morphology, 6 effect sizes from 3 study clusters and 115 participants showed a trivial and imprecise effect (g = 0.05, 95% CI −0.21 to 0.30, *p* = 0.718) (30, 33, 64), again with negligible heterogeneity. According to the GRADE assessment, the certainty of evidence was low for strength adaptations, low for muscle quantity/lean mass, and very low for CSA/direct morphology, primarily because of imprecision and sparse morphology data (Supplementary Tables S5, S6).

#### 3.4.2 Exploratory subgroup analyses

Exploratory subgroup analyses examined strength-test type, intervention duration, and polyphenol class, where sufficient study clusters were available (Table 4; Supplementary Table S7). These analyses were considered descriptive and hypothesis-generating. The conventional moderator test showed no difference among strength types (**P**_**diff**_ = 0.572). Although the isokinetic estimate was g = 0.43 (95% CI 0.03 to 0.83), it originated from one study cluster; therefore, its *P*-value and prediction interval were not reported. The CRVE-corrected moderator test yielded **P**_**diff**_ = 0.037, but was not interpreted because it involved only eight clusters, approximately three denominator degrees of freedom, and a single-cluster subgroup. For muscle quantity/lean mass, the catechin/flavanol-rich subgroup yielded g = 0.59 (95% CI 0.04 to 1.14; C = 4), but the polyphenol-class difference was not supported after CRVE correction (**P**_**diff**_ = 0.086). Intervention-duration analyses likewise provided no reliable subgroup differences. Overall, no subgroup analysis provided robust evidence of effect modification.

**Table 4:** Exploratory subgroup analyses for the primary outcome domains.

Outcome domain	Moderator	Subgroup	K	C	*N*	Hedges' g [95% CI]	*p*	
Strength adaptations	Strength type	Dynamic RM strength	14	5	141	0.32 [−0.10, 0.73]	0.139	0.572
		Isometric/dynamometer strength	4	3	84	0.04 [−0.35, 0.43]	0.833	
		Isokinetic strength	4	1	49	0.43 [0.03, 0.83]	NA	
	Population age group	Middle-aged/older participants	12	5	193	0.28 [−0.05, 0.60]	0.094	0.821
		Younger/adult participants	10	3	64	0.22 [−0.32, 0.76]	0.418	
	Polyphenol class	Catechin/flavanol-rich polyphenols	12	4	93	0.37 [−0.12, 0.86]	0.138	0.460
		Other polyphenols	10	4	164	0.14 [−0.13, 0.41]	0.301	
	Intervention duration	≤ 8 weeks	13	5	119	0.33 [−0.08, 0.74]	0.110	0.530
		>8 weeks	9	3	138	0.14 [−0.17, 0.46]	0.365	
Muscle quantity/lean mass	Population age group	Middle-aged/older participants	10	6	226	0.06 [−0.15, 0.28]	0.575	0.092
		Younger/adult participants	3	3	54	0.58 [−0.27, 1.43]	0.180	
	Polyphenol class	Catechin/flavanol-rich polyphenols	4	4	83	0.59 [0.04, 1.14]	0.035	0.023
		Other polyphenols	9	5	197	0.01 [−0.21, 0.23]	0.921	
	Intervention duration	≤ 8 weeks	4	4	90	0.32 [−0.14, 0.78]	0.170	0.299
		>8 weeks	9	5	190	0.07 [−0.16, 0.30]	0.544	

K denotes the number of effect sizes; C denotes the number of study clusters; N denotes participants. RM denotes repetition maximum. **P**_**diff**_ values are omnibus between-subgroup tests from exploratory three-level moderator models; blank cells indicate the same moderator-level test. All subgroup analyses were exploratory because no primary outcome domain included at least 10 study clusters. The isokinetic-strength subgroup contained one study cluster and was interpreted descriptively. The nominal polyphenol-class contrast for muscle quantity/lean mass was attenuated after CRVE small-sample correction.

#### 3.4.3 Sensitivity analyses

**Figure 7:**
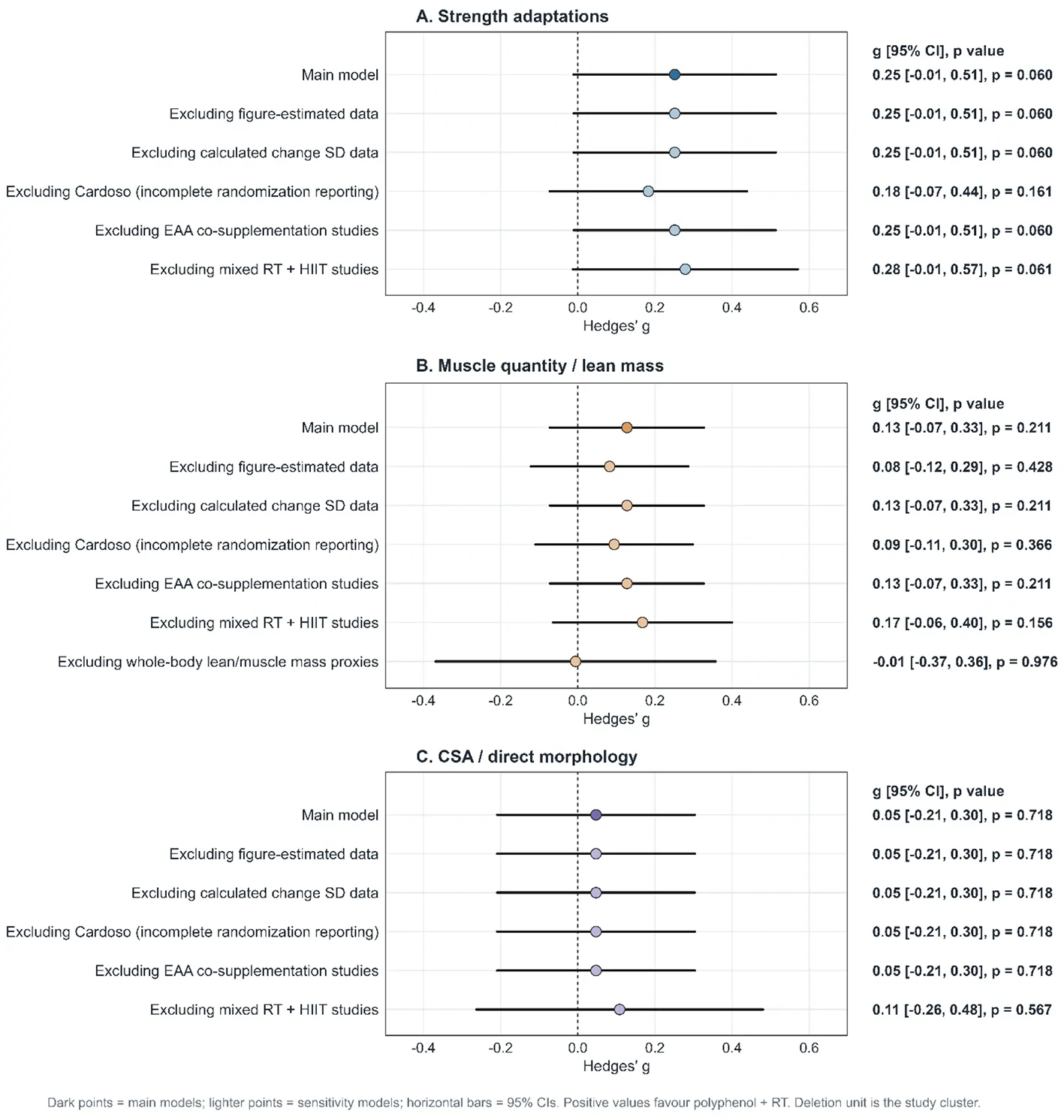
Pre-specified study-cluster deletion sensitivity analysis across the primary outcome domains.

**Figure 8:**
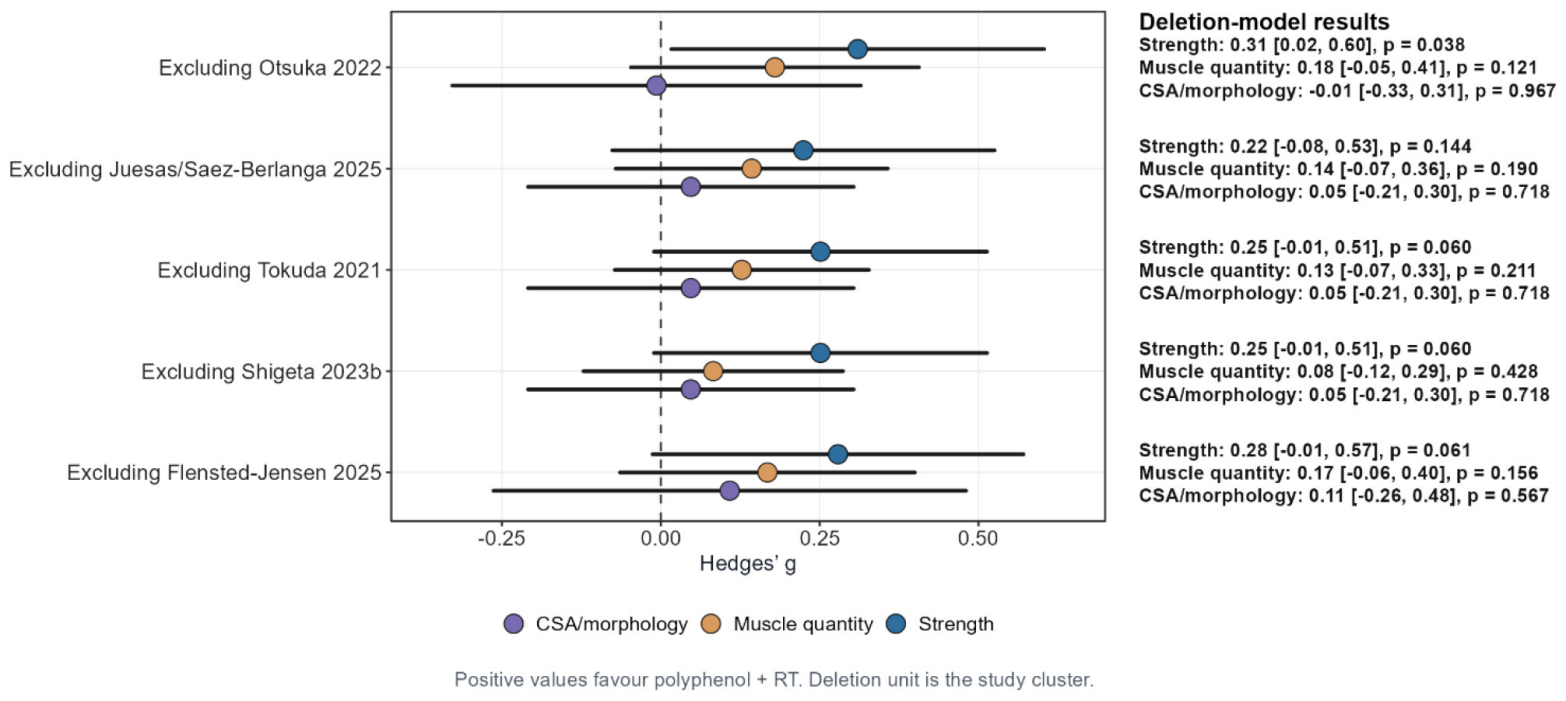
Category-based sensitivity analyses of pooled effects across the three primary outcome domains.

Complete leave-one-study-cluster-out analyses showed that no independently contributing cluster materially changed the overall interpretation (Supplementary Figure S11). Excluding Beyer or Otsuka produced nominal model-based strength signals, but neither remained statistically significant under CRVE (*P* = 0.059 and 0.086, respectively). Targeted deletion of clusters with pre-specified design or data concerns also did not alter the interpretation (Figure 7). Among pre-specified exclusion analyses, omitting Cardoso because the randomization procedures were incompletely reported, or omitting the mixed RT plus HIIT study, yielded statistically uncertain strength estimates (g = 0.18, 95% CI −0.07 to 0.44; and g = 0.28, 95% CI −0.01 to 0.57, respectively); muscle quantity/lean-mass estimates ranged from −0.01 to 0.17, and CSA/direct-morphology estimates remained close to zero (Figure 8). Recalculating missing change-score SDs using pre–post correlations of 0.30–0.70 produced strength estimates of g = 0.23–0.29, muscle quantity/lean-mass estimates of g = 0.12–0.14, and CSA/direct-morphology estimates of approximately g = 0.05; all confidence intervals crossed zero. The pooled-baseline-SD estimator produced similarly uncertain estimates at *r* = 0.50 (strength: g = 0.17; muscle quantity/lean mass: g = 0.08; CSA/direct morphology: g = 0.01), although the strength analysis was restricted to outcomes with available baseline SDs (Supplementary Figures S9, S10). CRVE-CR2 inference, assumed within-cluster sampling correlations of ρ = 0.30–0.90, and study-cluster aggregation did not alter the interpretation (Supplementary Figures S7, S8). Across ρ = 0.30–0.90, one-effect-per-cluster estimates ranged from g = 0.19 to 0.24 for strength, g = 0.15 to 0.19 for muscle quantity/lean mass, and approximately g = 0.05 for direct morphology; all confidence intervals crossed zero (Supplementary Table S8). Overall, no sensitivity analysis provided robust evidence requiring revision of the primary conclusion.

#### 3.4.4 Publication bias and small-study effects

**Figure 9:**
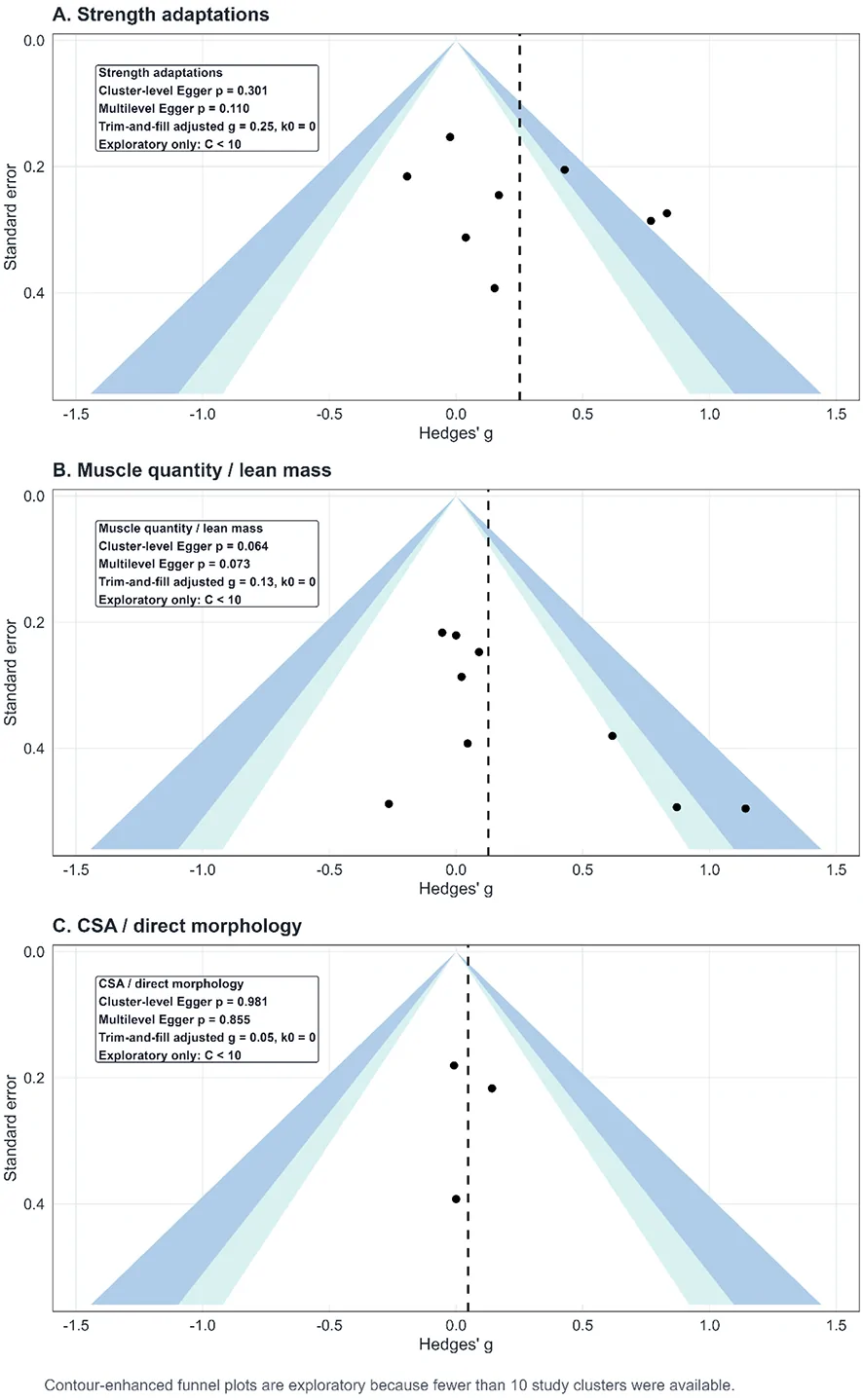
Contour-enhanced funnel plots for the three primary outcome domains. **(A)** Strength adaptations. **(B)** Muscle quantity/lean mass. **(C)** CSA/direct morphology.

Publication-bias analyses were exploratory because fewer than 10 study clusters contributed to each primary domain (Supplementary Table S4; Supplementary Figure S12). No consistent small-study-effect signal was identified for strength (Figure 9). For muscle quantity/lean mass, cluster-level and multilevel Egger tests produced borderline signals (*P* = 0.064 and 0.073), but these were not supported by CRVE (*P* = 0.163), and trim-and-fill imputed no missing studies. Publication bias could not be assessed reliably for CSA/direct morphology or fat mass because only three clusters were available; therefore, no trim-and-fill inference was made for these domains. These exploratory diagnostics did not provide evidence requiring revision of the primary conclusions, although small-study effects cannot be excluded.

## 4 Discussion

This systematic review and three-level meta-analysis did not establish that chronic polyphenol supplementation materially improves or attenuates resistance-training-induced adaptations in adults. Across 11 RCTs, strength adaptations showed a small positive but statistically uncertain estimate with low certainty, and both the confidence interval and prediction interval crossed the null. Muscle quantity/lean mass showed a small statistically uncertain estimate with low certainty, whereas CSA/direct morphology yielded an imprecise estimate close to zero with very low certainty because direct morphology data were sparse and limited to only three study clusters. Exploratory subgroup analyses did not provide robust evidence of effect modification by strength type, population age group, broad polyphenol class, or intervention duration, and the larger muscle quantity estimate for catechin/flavanol-rich interventions was not maintained after CRVE small-sample correction. Sensitivity analyses preserved the interpretation of the primary models, whereas publication-bias assessments were underpowered and could not reliably exclude publication bias because fewer than ten study clusters contributed to each domain. Overall, the available evidence is insufficient to establish either a reliable additive effect or a consistent attenuating effect on strength, muscle quantity/lean mass, or direct hypertrophic morphology.

The small strength-related signal is biologically plausible but should be interpreted cautiously. Strength gains after resistance training are not determined by hypertrophy alone; they also reflect neural drive, motor-unit behavior, intermuscular coordination, task learning, and testing specificity (66–68). Polyphenols may influence functional performance through recovery-related or vascular pathways, including fatigue tolerance, force recovery across repeated training sessions, and modulation of excessive oxidative or inflammatory stress (15, 69, 70). However, most included trials did not directly measure these neuromuscular, vascular, redox, or inflammatory mediators. The strength domain also combined dynamic RM (29–31, 34, 62), isometric/dynamometer-derived (33, 34, 64), and isokinetic outcomes (32, 61), which capture partly different functional properties. Therefore, the observed strength pattern is best viewed as a hypothesis-generating functional signal rather than direct evidence that polyphenols materially alter long-term strength adaptation.

The statistically uncertain estimate for muscle quantity/lean mass is consistent with current understanding of resistance-training hypertrophy. Muscle accretion is driven mainly by progressive mechanical loading, training volume, adequate energy and protein availability, and repeated stimulation of muscle protein remodeling (71–73). Unlike dietary protein or essential amino acids, polyphenols do not provide amino-acid substrate for myofibrillar protein accretion (74); any effect on muscle gain would therefore likely occur indirectly through modulation of training tolerance, inflammatory tone, vascular function, redox-sensitive signaling, or related regulatory pathways (75). Measurement level should also be considered. Lean mass, fat-free mass, regional lean mass, skeletal muscle mass, and appendicular indices are body-composition-derived proxies of muscle quantity, whereas MRI/CT-derived CSA, ultrasound muscle thickness, and fiber CSA more directly reflect localized morphology (76). Body-composition estimates derived from bioimpedance and prediction equations are sensitive to device characteristics, equation selection, reference methods, hydration status, and population-specific factors (77). Thus, the near-null estimate for muscle quantity/lean mass did not establish a consistent measurable difference in whole-body or regional muscle quantity, but does not exclude localized hypertrophic remodeling.

Given the sparse direct morphology data, the near-null CSA estimate should not be interpreted as evidence against localized hypertrophy (78). This limits conclusions about site-specific hypertrophy, muscle-thickness responses, and fiber-level remodeling. The finding also helps clarify the difference between acute recovery and chronic adaptation. Previous work suggests that flavonoid-rich or polyphenol-rich interventions may affect soreness, oxidative stress, inflammation, or short-term force recovery after exercise-induced muscle damage (14, 79). Yet these acute markers do not necessarily accumulate into greater muscle quantity or direct hypertrophy during progressive resistance training. Redox and inflammatory responses may be harmful when excessive, but they also participate in repair and remodeling processes (80). Polyphenols may also exert hormetic effects, whereby low-to-moderate phytochemical exposure activates endogenous antioxidant and cellular stress-response pathways, whereas excessive attenuation of exercise-induced redox signaling could theoretically impair adaptive signaling (17, 81). However, the included trials did not assess dose–response signaling or relevant molecular mediators; hormesis therefore remains a biologically plausible but untested explanation for the observed findings.

Inference is further constrained by the chemical and metabolic diversity of the tested interventions. Quercetin glycosides, curcumin, catechins, matcha, epicatechin, berry-derived polyphenols, green-tea products, and soy isoflavones differ substantially in structure, food matrix, intestinal absorption, phase-II conjugation, and gut microbial biotransformation. Class-level pooling was therefore necessary but conservative (75, 82–85). The catechin/flavanol-rich subgroup was used as a pragmatic exploratory category because it was the only related polyphenol subclass represented by at least four independent study clusters; this grouping was not intended to imply biological equivalence or superiority over other polyphenols. Several trials also included co-interventions or design features, including caffeine, essential amino acids, hypocaloric run-in periods, or concurrent HIIT, which limited polyphenol-specific attribution (6, 86). Reported administered dose and adherence records may not capture bioavailable or tissue-relevant exposure; future trials should therefore incorporate plasma or urinary metabolite profiling to support compound-specific interpretation (87). There is currently insufficient evidence to recommend chronic polyphenol supplementation as a strategy to augment resistance-training-induced gains in strength or muscle quantity/lean mass. The available evidence is likewise insufficient to establish a meaningful interference effect.

A methodological strength of this review was its conservative analytical strategy. Three-level random-effects models were used to account for dependent effect sizes arising from multiple outcomes, anatomical sites, dose arms, and overlapping reports, and CRVE, pre-specified sensitivity analyses, and leave-one-study-cluster-out analyses consistently supported the primary interpretation (88). However, exact within-study sampling covariances were unavailable, and the common-correlation sensitivity models could not represent source-specific dependence structures. The principal limitation was the size and resolution of the evidence base: each primary domain included fewer than ten independent clusters, and direct morphology included only three, limiting precision and preventing reliable moderator or dose–response analyses. Differences in polyphenol formulations, populations, training protocols, and measurement methods also restricted class-level generalizability. Shared controls, reconstructed change-score standard deviations, figure-extracted data, active co-supplementation, and mixed RT plus HIIT constrained polyphenol-specific attribution, although sensitivity analyses did not materially alter the interpretation. Incomplete reporting of habitual diet, adherence, training progression, and adverse events, together with unavailable full reports, introduced additional uncertainty. Future trials should use preregistered, compound-specific, placebo-controlled designs; verify biological exposure; standardize and monitor progressive RT; control relevant dietary factors; prioritize direct morphology; and report complete change-score data.

## 5 Conclusion

This systematic review and three-level meta-analysis did not establish that chronic polyphenol supplementation reliably modifies resistance-training-induced adaptations in strength, muscle quantity/lean mass, or direct muscle morphology in adults. Strength showed a small, directionally positive but statistically uncertain estimate, whereas estimates for muscle quantity/lean mass and direct morphology were also uncertain. Overall, these findings represent insufficient evidence of a consistent effect rather than evidence that all polyphenol interventions are ineffective. They neither support recommending chronic polyphenol supplementation to augment resistance-training adaptations nor permit firm conclusions regarding possible attenuation or context-specific effects. Future trials should isolate defined polyphenol exposures, verify biological exposure, standardize and monitor progressive resistance training, control relevant dietary factors, and prioritize direct assessments of muscle morphology.

## Data Availability

The original contributions presented in the study are included in the article/Supplementary material, further inquiries can be directed to the corresponding author.
