# Supplementary material for: Does chronic polyphenol supplementation modify resistance training-induced strength and muscle morphology adaptations in adults? A systematic review and multilevel meta-analysis of randomized controlled trials
Source: Front Nutr. 2026 Aug 28;13:1906384. doi: 10.3389/fnut.2026.1906384 (PMC13562237; doi:10.3389/fnut.2026.1906384)
Supplement: Supplementary file 1 [file Table_1.DOCX]

# 1 Supplementary Table S1

Table S1 Number of hits for the complete search strategy in the databases

| Database | Complete Search Strategy | Hits May 21, 2026 |
| --- | --- | --- |
| PubMed | (("Polyphenols"[MeSH Terms] OR "Flavonoids"[MeSH Terms] OR "Phenols"[MeSH Terms] OR "Anthocyanins"[MeSH Terms] OR "Catechin"[MeSH Terms] OR "Quercetin"[MeSH Terms] OR "Isoflavones"[MeSH Terms] OR "Resveratrol"[MeSH Terms] OR "Curcumin"[MeSH Terms] OR "Tannins"[MeSH Terms] OR "Proanthocyanidins"[MeSH Terms] OR "Lignans"[MeSH Terms] OR "Tea"[MeSH Terms] OR "polyphenol*"[Title/Abstract] OR "polyphenolic*"[Title/Abstract] OR "polyphenol-rich"[Title/Abstract] OR "flavonoid*"[Title/Abstract] OR "flavonoid-rich"[Title/Abstract] OR "flavon*"[Title/Abstract] OR "flavanol*"[Title/Abstract] OR "flavan 3 ol*"[Title/Abstract] OR "flavonol*"[Title/Abstract] OR "flavone*"[Title/Abstract] OR "flavanone*"[Title/Abstract] OR "isoflavone*"[Title/Abstract] OR "phenolic*"[Title/Abstract] OR "phenolic acid*"[Title/Abstract] OR "anthocyanin*"[Title/Abstract] OR "anthocyanidin*"[Title/Abstract] OR "catechin*"[Title/Abstract] OR "epicatechin"[Title/Abstract] OR "epigallocatechin gallate"[Title/Abstract] OR "EGCG"[Title/Abstract] OR "proanthocyanidin*"[Title/Abstract] OR "tannin*"[Title/Abstract] OR "ellagitannin*"[Title/Abstract] OR "ellagic acid"[Title/Abstract] OR "punicalagin"[Title/Abstract] OR "urolithin*"[Title/Abstract] OR "stilbene*"[Title/Abstract] OR "Resveratrol"[Title/Abstract] OR "pterostilbene"[Title/Abstract] OR "Quercetin"[Title/Abstract] OR "rutin"[Title/Abstract] OR "kaempferol"[Title/Abstract] OR "myricetin"[Title/Abstract] OR "fisetin"[Title/Abstract] OR "apigenin"[Title/Abstract] OR "luteolin"[Title/Abstract] OR "hesperidin"[Title/Abstract] OR "hesperetin"[Title/Abstract] OR "naringin"[Title/Abstract] OR "naringenin"[Title/Abstract] OR "genistein"[Title/Abstract] OR "daidzein"[Title/Abstract] OR "soy isoflavone*"[Title/Abstract] OR "soybean isoflavone*"[Title/Abstract] OR "Curcumin"[Title/Abstract] OR "curcuminoid*"[Title/Abstract] OR "turmeric"[Title/Abstract] OR "chlorogenic acid"[Title/Abstract] OR "caffeic acid"[Title/Abstract] OR "ferulic acid"[Title/Abstract] OR "gallic acid"[Title/Abstract] OR "green tea"[Title/Abstract] OR "tea catechin*"[Title/Abstract] OR "green tea extract"[Title/Abstract] OR "Camellia sinensis"[Title/Abstract] OR "matcha"[Title/Abstract] OR "cocoa"[Title/Abstract] OR "cacao"[Title/Abstract] OR "cocoa flavanol*"[Title/Abstract] OR "cocoa polyphenol*"[Title/Abstract] OR "pomegranate"[Title/Abstract] OR "pomegranate extract"[Title/Abstract] OR "tart cherry"[Title/Abstract] OR "Montmorency"[Title/Abstract] OR "cherry juice"[Title/Abstract] OR "cherry extract"[Title/Abstract] OR "blueberry"[Title/Abstract] OR "bilberry"[Title/Abstract] OR "berry"[Title/Abstract] OR "berries"[Title/Abstract] OR "berry extract*"[Title/Abstract] OR "blackcurrant"[Title/Abstract] OR "cranberry"[Title/Abstract] OR "chokeberry"[Title/Abstract] OR "aronia"[Title/Abstract] OR "grape"[Title/Abstract] OR "grapes"[Title/Abstract] OR "grape seed"[Title/Abstract] OR "grape juice"[Title/Abstract] OR "grape extract"[Title/Abstract] OR "Vitis vinifera"[Title/Abstract] OR "apple polyphenol*"[Title/Abstract] OR "hydroxytyrosol"[Title/Abstract] OR "oleuropein"[Title/Abstract] OR "olive polyphenol*"[Title/Abstract] OR "olive leaf extract"[Title/Abstract] OR "phlorotannin*"[Title/Abstract] OR "seaweed polyphenol*"[Title/Abstract]) AND ("Resistance Training"[MeSH Terms] OR "weight lifting"[MeSH Terms] OR "Resistance Training"[Title/Abstract] OR "resistance train*"[Title/Abstract] OR "resistance exercise*"[Title/Abstract] OR "resistive exercise*"[Title/Abstract] OR "resistive training"[Title/Abstract] OR "progressive resistance training"[Title/Abstract] OR "progressive resistive exercise*"[Title/Abstract] OR "progressive resistance exercise*"[Title/Abstract] OR "strength training"[Title/Abstract] OR "strength train*"[Title/Abstract] OR "strength exercise*"[Title/Abstract] OR "weight training"[Title/Abstract] OR "weight train*"[Title/Abstract] OR "weight lifting"[Title/Abstract] OR "weightlifting"[Title/Abstract] OR "weight lifting"[Title/Abstract] OR "weight lift*"[Title/Abstract] OR "resistance type exercise*"[Title/Abstract] OR "resistance-type training"[Title/Abstract] OR "load training"[Title/Abstract] OR "loaded exercise*"[Title/Abstract] OR "bodyweight training"[Title/Abstract] OR "calisthenic*"[Title/Abstract] OR "power training"[Title/Abstract] OR "high-velocity resistance training"[Title/Abstract] OR "squat*"[Title/Abstract] OR "leg press"[Title/Abstract] OR "bench press"[Title/Abstract] OR "knee extension"[Title/Abstract] OR "chest press"[Title/Abstract] OR "eccentric training"[Title/Abstract] OR "eccentric exercise*"[Title/Abstract] OR "isometric training"[Title/Abstract] OR "isokinetic training"[Title/Abstract] OR "plyometric training"[Title/Abstract] OR "plyometric exercise*"[Title/Abstract]) AND ("randomized controlled trial"[Publication Type] OR "controlled clinical trial"[Publication Type] OR "clinical trial"[Publication Type] OR "randomized"[Title/Abstract] OR "randomised"[Title/Abstract] OR "randomly"[Title/Abstract] OR "random*"[Title/Abstract] OR "placebo"[Title/Abstract] OR "placebo*"[Title/Abstract] OR "trial"[Title/Abstract] OR "groups"[Title/Abstract] OR "allocation"[Title/Abstract] OR "double-blind"[Title/Abstract] OR "double-blind"[Title/Abstract] OR "single-blind"[Title/Abstract] OR "single-blind"[Title/Abstract] OR "crossover"[Title/Abstract] OR "cross-over"[Title/Abstract] OR "parallel-group"[Title/Abstract] OR "parallel-group"[Title/Abstract])) NOT ("animals"[MeSH Terms] NOT "humans"[MeSH Terms]) | 287 |
| Web of Science | TS=(polyphenol* OR polyphenolic* OR "polyphenol-rich" OR flavonoid* OR "flavonoid-rich" OR flavon* OR flavanol* OR "flavan-3-ol*" OR flavonol* OR flavone* OR flavanone* OR isoflavone* OR phenolic* OR "phenolic acid*" OR anthocyanin* OR anthocyanidin* OR catechin* OR epicatechin OR "epigallocatechin gallate" OR EGCG OR proanthocyanidin* OR tannin* OR ellagitannin* OR "ellagic acid" OR punicalagin OR urolithin* OR stilbene* OR resveratrol OR pterostilbene OR quercetin OR rutin OR kaempferol OR myricetin OR fisetin OR apigenin OR luteolin OR hesperidin OR hesperetin OR naringin OR naringenin OR genistein OR daidzein OR "soy isoflavone*" OR "soybean isoflavone*" OR curcumin OR curcuminoid* OR turmeric OR "chlorogenic acid" OR "caffeic acid" OR "ferulic acid" OR "gallic acid" OR "green tea" OR "tea catechin*" OR "green tea extract" OR "Camellia sinensis" OR matcha OR cocoa OR cacao OR "cocoa flavanol*" OR "cocoa polyphenol*" OR pomegranate OR "pomegranate extract" OR "tart cherry" OR Montmorency OR "cherry juice" OR "cherry extract" OR blueberry OR bilberry OR berry OR berries OR "berry extract*" OR blackcurrant OR cranberry OR chokeberry OR aronia OR grape OR grapes OR "grape seed" OR "grape juice" OR "grape extract" OR "Vitis vinifera" OR "apple polyphenol*" OR hydroxytyrosol OR oleuropein OR "olive polyphenol*" OR "olive leaf extract" OR phlorotannin* OR "seaweed polyphenol*")  AND TS=("resistance training" OR "resistance train*" OR "resistance exercise*" OR "resistive exercise*" OR "resistive training" OR "progressive resistance training" OR "progressive resistive exercise*" OR "progressive resistance exercise*" OR "strength training" OR "strength train*" OR "strength exercise*" OR "weight training" OR "weight train*" OR "weight lifting" OR weightlifting OR "weight-lifting" OR "weight lift*" OR "resistance-type exercise*" OR "resistance-type training" OR "load training" OR "loaded exercise*" OR "bodyweight training" OR calisthenic* OR "power training" OR "high-velocity resistance training" OR squat* OR "leg press" OR "bench press" OR "knee extension" OR "chest press" OR "eccentric training" OR "eccentric exercise*" OR "isometric training" OR "isokinetic training" OR "plyometric training" OR "plyometric exercise*")  AND TS=(randomized OR randomised OR randomly OR random* OR placebo OR placebo* OR trial OR groups OR allocation OR "double blind" OR "double-blind" OR "single blind" OR "single-blind" OR crossover OR "cross-over" OR "parallel group" OR "parallel-group") | 333 |
| Cochrane library | #1: MeSH descriptor: [Polyphenols] explode all trees  #2: MeSH descriptor: [Flavonoids] explode all trees  #3: MeSH descriptor: [Phenols] explode all trees  #4: MeSH descriptor: [Anthocyanins] explode all trees  #5: MeSH descriptor: [Catechin] explode all trees  #6: MeSH descriptor: [Quercetin] explode all trees  #7: MeSH descriptor: [Isoflavones] explode all trees  #8: MeSH descriptor: [Resveratrol] explode all trees  #9: MeSH descriptor: [Curcumin] explode all trees  #10: MeSH descriptor: [Tannins] explode all trees  #11: (polyphenol* OR polyphenolic* OR "polyphenol-rich" OR flavonoid* OR "flavonoid-rich" OR flavon* OR flavanol* OR (flavan NEXT 3 NEXT ol*) OR flavonol* OR flavone* OR flavanone* OR isoflavone* OR phenolic* OR (phenolic NEXT acid*) OR anthocyanin* OR anthocyanidin* OR catechin* OR epicatechin OR "epigallocatechin gallate" OR EGCG OR proanthocyanidin* OR tannin* OR ellagitannin* OR "ellagic acid" OR punicalagin OR urolithin* OR stilbene* OR resveratrol OR pterostilbene OR quercetin OR rutin OR kaempferol OR myricetin OR fisetin OR apigenin OR luteolin OR hesperidin OR hesperetin OR naringin OR naringenin OR genistein OR daidzein OR (soy NEXT isoflavone*) OR (soybean NEXT isoflavone*) OR curcumin OR curcuminoid* OR turmeric OR "chlorogenic acid" OR "caffeic acid" OR "ferulic acid" OR "gallic acid" OR "green tea" OR (tea NEXT catechin*) OR "green tea extract" OR "Camellia sinensis" OR matcha OR cocoa OR cacao OR (cocoa NEXT flavanol*) OR (cocoa NEXT polyphenol*) OR pomegranate OR "pomegranate extract" OR "tart cherry" OR Montmorency OR "cherry juice" OR "cherry extract" OR blueberry OR bilberry OR berry OR berries OR (berry NEXT extract*) OR blackcurrant OR cranberry OR chokeberry OR aronia OR grape OR grapes OR "grape seed" OR "grape juice" OR "grape extract" OR "Vitis vinifera" OR (apple NEXT polyphenol*) OR hydroxytyrosol OR oleuropein OR (olive NEXT polyphenol*) OR "olive leaf extract" OR phlorotannin* OR (seaweed NEXT polyphenol*)):ti,ab,kw  #12: #1 OR #2 OR #3 OR #4 OR #5 OR #6 OR #7 OR #8 OR #9 OR #10 OR #11  #13: MeSH descriptor: [Resistance Training] explode all trees  #14: MeSH descriptor: [Weight Lifting] explode all trees  #15: ("resistance training" OR (resistance NEXT train*) OR (resistance NEXT exercise*) OR (resistive NEXT exercise*) OR "resistive training" OR "progressive resistance training" OR (progressive NEXT resistive NEXT exercise*) OR (progressive NEXT resistance NEXT exercise*) OR "strength training" OR (strength NEXT train*) OR (strength NEXT exercise*) OR "weight training" OR (weight NEXT train*) OR "weight lifting" OR weightlifting OR "weight-lifting" OR (weight NEXT lift*) OR ((resistance-type OR (resistance NEXT type)) NEXT exercise*) OR "resistance-type training" OR "load training" OR (loaded NEXT exercise*) OR "bodyweight training" OR calisthenic* OR "power training" OR "high-velocity resistance training" OR squat* OR "leg press" OR "bench press" OR "knee extension" OR "chest press" OR "eccentric training" OR (eccentric NEXT exercise*) OR "isometric training" OR "isokinetic training" OR "plyometric training" OR (plyometric NEXT exercise*)):ti,ab,kw  #16: #13 OR #14 OR #15  #17: #12 AND #16 | 347 |
| Embase | ('polyphenol'/exp OR 'flavonoid'/exp OR 'phenol derivative'/exp OR 'anthocyanin'/exp OR 'catechin'/exp OR 'quercetin'/exp OR 'isoflavone derivative'/exp OR 'resveratrol'/exp OR 'curcumin'/exp OR 'tannin derivative'/exp OR 'proanthocyanidin'/exp OR 'lignan'/exp OR 'tea'/exp OR 'polyphenol*':ti,ab,kw OR 'polyphenolic*':ti,ab,kw OR 'polyphenol-rich':ti,ab,kw OR 'flavonoid*':ti,ab,kw OR 'flavonoid-rich':ti,ab,kw OR 'flavon*':ti,ab,kw OR 'flavanol*':ti,ab,kw OR 'flavan 3 ol*':ti,ab,kw OR 'flavonol*':ti,ab,kw OR 'flavone*':ti,ab,kw OR 'flavanone*':ti,ab,kw OR 'isoflavone*':ti,ab,kw OR 'phenolic*':ti,ab,kw OR 'phenolic acid*':ti,ab,kw OR 'anthocyanin*':ti,ab,kw OR 'anthocyanidin*':ti,ab,kw OR 'catechin*':ti,ab,kw OR 'epicatechin':ti,ab,kw OR 'epigallocatechin gallate':ti,ab,kw OR 'egcg':ti,ab,kw OR 'proanthocyanidin*':ti,ab,kw OR 'tannin*':ti,ab,kw OR 'ellagitannin*':ti,ab,kw OR 'ellagic acid':ti,ab,kw OR 'punicalagin':ti,ab,kw OR 'urolithin*':ti,ab,kw OR 'stilbene*':ti,ab,kw OR 'resveratrol':ti,ab,kw OR 'pterostilbene':ti,ab,kw OR 'quercetin':ti,ab,kw OR 'rutin':ti,ab,kw OR 'kaempferol':ti,ab,kw OR 'myricetin':ti,ab,kw OR 'fisetin':ti,ab,kw OR 'apigenin':ti,ab,kw OR 'luteolin':ti,ab,kw OR 'hesperidin':ti,ab,kw OR 'hesperetin':ti,ab,kw OR 'naringin':ti,ab,kw OR 'naringenin':ti,ab,kw OR 'genistein':ti,ab,kw OR 'daidzein':ti,ab,kw OR 'soy isoflavone*':ti,ab,kw OR 'soybean isoflavone*':ti,ab,kw OR 'curcumin':ti,ab,kw OR 'curcuminoid*':ti,ab,kw OR 'turmeric':ti,ab,kw OR 'chlorogenic acid':ti,ab,kw OR 'caffeic acid':ti,ab,kw OR 'ferulic acid':ti,ab,kw OR 'gallic acid':ti,ab,kw OR 'green tea':ti,ab,kw OR 'tea catechin*':ti,ab,kw OR 'green tea extract':ti,ab,kw OR 'camellia sinensis':ti,ab,kw OR 'matcha':ti,ab,kw OR 'cocoa':ti,ab,kw OR 'cacao':ti,ab,kw OR 'cocoa flavanol*':ti,ab,kw OR 'cocoa polyphenol*':ti,ab,kw OR 'pomegranate':ti,ab,kw OR 'pomegranate extract':ti,ab,kw OR 'tart cherry':ti,ab,kw OR 'montmorency':ti,ab,kw OR 'cherry juice':ti,ab,kw OR 'cherry extract':ti,ab,kw OR 'blueberry':ti,ab,kw OR 'bilberry':ti,ab,kw OR 'berry':ti,ab,kw OR 'berries':ti,ab,kw OR 'berry extract*':ti,ab,kw OR 'blackcurrant':ti,ab,kw OR 'cranberry':ti,ab,kw OR 'chokeberry':ti,ab,kw OR 'aronia':ti,ab,kw OR 'grape':ti,ab,kw OR 'grapes':ti,ab,kw OR 'grape seed':ti,ab,kw OR 'grape juice':ti,ab,kw OR 'grape extract':ti,ab,kw OR 'vitis vinifera':ti,ab,kw OR 'apple polyphenol*':ti,ab,kw OR 'hydroxytyrosol':ti,ab,kw OR 'oleuropein':ti,ab,kw OR 'olive polyphenol*':ti,ab,kw OR 'olive leaf extract':ti,ab,kw OR 'phlorotannin*':ti,ab,kw OR 'seaweed polyphenol*':ti,ab,kw) AND ('resistance training'/exp OR 'weight lifting'/exp OR 'resistance training':ti,ab,kw OR 'resistance train*':ti,ab,kw OR 'resistance exercise*':ti,ab,kw OR 'resistive exercise*':ti,ab,kw OR 'resistive training':ti,ab,kw OR 'progressive resistance training':ti,ab,kw OR 'progressive resistive exercise*':ti,ab,kw OR 'progressive resistance exercise*':ti,ab,kw OR 'strength training':ti,ab,kw OR 'strength train*':ti,ab,kw OR 'strength exercise*':ti,ab,kw OR 'weight training':ti,ab,kw OR 'weight train*':ti,ab,kw OR 'weightlifting':ti,ab,kw OR 'weight lifting':ti,ab,kw OR 'weight lift*':ti,ab,kw OR 'resistance type exercise*':ti,ab,kw OR 'resistance-type training':ti,ab,kw OR 'load training':ti,ab,kw OR 'loaded exercise*':ti,ab,kw OR 'bodyweight training':ti,ab,kw OR 'calisthenic*':ti,ab,kw OR 'power training':ti,ab,kw OR 'high-velocity resistance training':ti,ab,kw OR 'squat*':ti,ab,kw OR 'leg press':ti,ab,kw OR 'bench press':ti,ab,kw OR 'knee extension':ti,ab,kw OR 'chest press':ti,ab,kw OR 'eccentric training':ti,ab,kw OR 'eccentric exercise*':ti,ab,kw OR 'isometric training':ti,ab,kw OR 'isokinetic training':ti,ab,kw OR 'plyometric training':ti,ab,kw OR 'plyometric exercise*':ti,ab,kw) AND ('randomized controlled trial':it OR 'controlled clinical trial':it OR 'clinical trial':it OR 'randomized':ti,ab,kw OR 'randomised':ti,ab,kw OR 'randomly':ti,ab,kw OR 'random*':ti,ab,kw OR 'placebo':ti,ab,kw OR 'placebo*':ti,ab,kw OR 'trial':ti,ab,kw OR 'groups':ti,ab,kw OR 'allocation':ti,ab,kw OR 'double-blind':ti,ab,kw OR 'single-blind':ti,ab,kw OR 'crossover':ti,ab,kw OR 'cross-over':ti,ab,kw OR 'parallel-group':ti,ab,kw) NOT ('animal'/exp NOT 'human'/exp) | 497 |
| Scopus | TITLE-ABS-KEY ( polyphenol* OR polyphenolic* OR "polyphenol-rich" OR flavonoid* OR "flavonoid-rich" OR flavon* OR flavanol* OR "flavan-3-ol*" OR flavonol* OR flavone* OR flavanone* OR isoflavone* OR phenolic* OR "phenolic acid*" OR anthocyanin* OR anthocyanidin* OR catechin* OR epicatechin OR "epigallocatechin gallate" OR EGCG OR proanthocyanidin* OR tannin* OR ellagitannin* OR "ellagic acid" OR punicalagin OR urolithin* OR stilbene* OR resveratrol OR pterostilbene OR quercetin OR rutin OR kaempferol OR myricetin OR fisetin OR apigenin OR luteolin OR hesperidin OR hesperetin OR naringin OR naringenin OR genistein OR daidzein OR "soy isoflavone*" OR "soybean isoflavone*" OR curcumin OR curcuminoid* OR turmeric OR "chlorogenic acid" OR "caffeic acid" OR "ferulic acid" OR "gallic acid" OR "green tea" OR "tea catechin*" OR "green tea extract" OR "Camellia sinensis" OR matcha OR cocoa OR cacao OR "cocoa flavanol*" OR "cocoa polyphenol*" OR pomegranate OR "pomegranate extract" OR "tart cherry" OR Montmorency OR "cherry juice" OR "cherry extract" OR blueberry OR bilberry OR berry OR berries OR "berry extract*" OR blackcurrant OR cranberry OR chokeberry OR aronia OR grape OR grapes OR "grape seed" OR "grape juice" OR "grape extract" OR "Vitis vinifera" OR "apple polyphenol*" OR hydroxytyrosol OR oleuropein OR "olive polyphenol*" OR "olive leaf extract" OR phlorotannin* OR "seaweed polyphenol*" ) AND TITLE-ABS-KEY ( "resistance training" OR "resistance train*" OR "resistance exercise*" OR "resistive exercise*" OR "resistive training" OR "progressive resistance training" OR "progressive resistive exercise*" OR "progressive resistance exercise*" OR "strength training" OR "strength train*" OR "strength exercise*" OR "weight training" OR "weight train*" OR "weight lifting" OR weightlifting OR "weight-lifting" OR "weight lift*" OR "resistance-type exercise*" OR "resistance-type training" OR "load training" OR "loaded exercise*" OR "bodyweight training" OR calisthenic* OR "power training" OR "high-velocity resistance training" OR squat* OR "leg press" OR "bench press" OR "knee extension" OR "chest press" OR "eccentric training" OR "eccentric exercise*" OR "isometric training" OR "isokinetic training" OR "plyometric training" OR "plyometric exercise*" ) AND TITLE-ABS-KEY ( randomized OR randomised OR randomly OR random* OR placebo OR placebo* OR trial OR groups OR allocation OR "double blind" OR "double-blind" OR "single blind" OR "single-blind" OR crossover OR "cross-over" OR "parallel group" OR "parallel-group" ) AND ( LIMIT-TO ( DOCTYPE , "ar" ) OR LIMIT-TO ( DOCTYPE , "cp" ) ) | 298 |

# 2 Supplementary Table S2

Table S2 Risk of bias domains and overall judgements

| Study | D1 | D2 | D3 | D4 | D5 | Overall |
| --- | --- | --- | --- | --- | --- | --- |
| Flensted-Jensen et al. 2025 | Low | Low | Some concerns | Low | Low | Some concerns |
| Juesas et al. 2025 | Low | Low | Low | Low | Low | Low |
| Saez-Berlanga et al. 2025 | Low | Low | Low | Low | Low | Low |
| Nishikawa et al. 2025 | Low | Low | Low | Low | Low | Low |
| Shigeta et al. 2023 | Some concerns | Some concerns | Low | Low | Low | Some concerns |
| Otsuka et al. 2022 | Low | Low | Low | Low | Low | Low |
| Tokuda & Mori 2021 | Some concerns | Some concerns | Low | Low | Low | Some concerns |
| Mafi et al. 2018 | Some concerns | Low | Low | Low | Low | Some concerns |
| Beyer et al. 2017 | Some concerns | Low | Some concerns | Low | Low | Some concerns |
| Cardoso et al. 2013 | Some concerns | Low | Low | Low | Low | Some concerns |
| Orsatti et al. 2010 | Low | Low | Low | Low | Low | Low |

Note: D1 = Randomization process; D2 = Deviations from the intended interventions; D3 = Missing outcome data; D4 = Measurement of the outcome; D5 = Selection of the reported result.

# 3 Study selection consistency and Cohen’s κ

**Title/abstract screening (records screened = 756)**

Independent 2×2 table (NS vs CBW): both include = 34; NS include / CBW exclude = 1; NS exclude / CBW include = 2; both exclude = 719 (total n = 756; reports sought for retrieval a + b + c = 37, consistent with the PRISMA flowchart).

Observed agreement: Po = (34 + 719) / 756 = 0.9960.

Expected agreement: Pe = [(35 × 36) + (721 × 720)] / 756² ≈ 0.9105.

Cohen’s κ = (Po − Pe) / (1 − Pe) ≈ 0.956.

Approximate standard error: SE ≈ 0.026.

95% CI: κ ≈ 0.956, 95% CI ≈ 0.906–1.006 (upper bound truncated at 1.00 as customary when the normal approximation slightly exceeds 1).

Interpretation (Landis & Koch): **κ ≈ 0.96, indicating almost perfect agreement.**

**Full-text screening (reports assessed = 30)**

Independent 2×2 table (NS vs CBW): both include = 9; NS include / CBW exclude = 1; NS exclude / CBW include = 1; both exclude = 19 (total n = 30; reports judged as eligible by at least one reviewer a + b + c = 11). After discussion/adjudication, 11 reports were finally included and 19 were excluded, consistent with the PRISMA flowchart.

Observed agreement: Po = (9 + 19) / 30 = 0.9333.

Expected agreement: Pe = [(10 × 10) + (20 × 20)] / 30² ≈ 0.5556.

Cohen’s κ = (Po − Pe) / (1 − Pe) ≈ 0.850.

Approximate standard error: SE ≈ 0.102.

95% CI: κ ≈ 0.850, 95% CI ≈ 0.649–1.051 (upper bound truncated at 1.00 as customary when the normal approximation slightly exceeds 1).

Interpretation (Landis & Koch): **κ ≈ 0.85, indicating almost perfect agreement.**

# 4 Supplementary Table S3

Table S3 Inter-Rater Agreement Results for Each ROB2 Domain

| Domain | Final consensus distribution | Simple agreement | Cohen’s κ | Interpretation |
| --- | --- | --- | --- | --- |
| D1: Randomization process | Low = 6; Some concerns = 5 | 81.8% (9/11) | 0.677 | Substantial |
| D2: Deviations from intended interventions | Low = 9; Some concerns = 2 | 90.9% (10/11) | 0.744 | Substantial |
| D3: Missing outcome data | Low = 9; Some concerns = 2 | 100.0% (11/11) | 1.000 | Almost perfect |
| D4: Measurement of the outcome | Low = 11 | 100.0% (11/11) | Not separately informative | Complete agreement |
| D5: Selection of the reported result | Low = 11 | 100.0% (11/11) | Not separately informative | Complete agreement |

**Table notes:** RoB 2 = Risk of Bias 2 tool. Cohen’s κ was calculated for domains with rating variability. For D4 and D5, all reports were assigned the same category by both reviewers; therefore, simple agreement was 100%, and κ was not separately informative. Agreement strength was interpreted according to Landis and Koch.

# 5 PEDro item-level agreement

PEDro item-level agreement was calculated using the 10 scored PEDro items, excluding item 1 because it is not counted toward the PEDro total score. Across 11 reports, 110 scored item-level judgments were evaluated.

Independent 2×2 table for PEDro scored items (NS vs CBW): both assigned “1” (Yes) = 82; NS assigned “1” / CBW assigned “0” = 2; NS assigned “0” / CBW assigned “1” = 2; both assigned “0” (No) = 24. Total n = 110 scored PEDro items. After discussion/adjudication, the final consensus included 86 “1” scores and 24 “0” scores, consistent with the final PEDro summary table.

Observed agreement: Po = (82 + 24) / 110 = 0.9636.

Expected agreement: Pe = [(84 × 84) + (26 × 26)] / 110² = 7732 / 12100 ≈ 0.6390.

Cohen’s κ = (Po − Pe) / (1 − Pe) ≈ 0.899.

Approximate standard error: SE ≈ 0.050.

95% CI: κ ≈ 0.899, 95% CI ≈ 0.802–0.996.

Interpretation (Landis & Koch): κ ≈ 0.90, indicating almost perfect agreement.

# 6 Supplementary Table S4

Table S4 Publication-bias and small-study-effect diagnostics

| Domain | C | Cluster-level Egger p | Multilevel Egger p | CRVE Egger p | Trim-and-fill k0 | Adjusted g [95% CI] | Interpretation |
| --- | --- | --- | --- | --- | --- | --- | --- |
| Strength adaptations | 8 | 0.301 | 0.110 | 0.146 | 0 | 0.25 [-0.01, 0.51] | Exploratory; fewer than 10 study clusters |
| Muscle quantity / lean mass | 9 | 0.064 | 0.073 | 0.163 | 0 | 0.13 [-0.07, 0.33] | Borderline asymmetry signal; CRVE non-significant; no trim-and-fill imputation |
| CSA / direct morphology | 3 | Not reliably assessable | Not reliably assessable | Not reliably assessable | Not performed | Not reported | Too few clusters for reliable assessment |

**Table Notes:** C denotes the number of study clusters. Egger p values were derived from cluster-level, multilevel, and CRVE-based Egger tests, respectively. Trim-and-fill k0 denotes the number of imputed missing studies. Adjusted g represents the trim-and-fill adjusted effect estimate. All publication-bias diagnostics were exploratory because fewer than ten study clusters contributed to each primary outcome domain. CRVE, cluster-robust variance estimation; CSA, cross-sectional area; CI, confidence interval. Publication bias was not reliably assessable for CSA / direct morphology (C = 3), and no trim-and-fill inference was made for that domain.

# 7 Supplementary Table S5

Table S5 GRADE summary of findings

| Outcome | Evidence base | Effect | 95% PI | Heterogeneity | Risk-of-bias sensitivity | Certainty |
| --- | --- | --- | --- | --- | --- | --- |
| Strength adaptations | K = 22; C = 8; N = 257 | g = 0.25 [−0.01, 0.51]; p = 0.060 | −0.36 to 0.86 | I²within = 0.0%; I²between = 34.5% | g = 0.18 [−0.07, 0.44]; p = 0.161 | Low |
| Muscle quantity / lean mass | K = 13; C = 9; N = 280 | g = 0.13 [−0.07, 0.33]; p = 0.211 | −0.07 to 0.33 | I²within = 0.0%; I²between = 0.0% | g = 0.09 [−0.11, 0.30]; p = 0.366 | Low |
| CSA / direct morphology | K = 6; C = 3; N = 115 | g = 0.05 [−0.21, 0.30]; p = 0.718 | −0.21 to 0.30 | I²within = 0.0%; I²between = 0.0% | No high-risk cluster present | Very low |

**Table Notes:** K denotes the number of effect sizes; C denotes the number of study clusters; N denotes the number of unique participants contributing to the corresponding outcome domain; each independent participant sample was counted once, irrespective of the number of effect sizes contributed. Effect values are presented as Hedges’ g [95% CI]. PI denotes prediction interval. Positive values favor polyphenol supplementation plus resistance training. Certainty was downgraded for class-level indirectness and imprecision; direct morphology was additionally limited by sparse data. Certainty was assessed using the GRADE framework.

# 8 Supplementary Table S6

Table S6 GRADE domain-level judgments

| Outcome | Risk of bias | Inconsistency | Indirectness | Imprecision | Publication bias | Overall certainty | Basis for downgrading |
| --- | --- | --- | --- | --- | --- | --- | --- |
| Strength adaptations | Not serious | Not serious | Serious | Serious | Undetected; exploratory | Low | Downgraded for serious class-level indirectness and serious imprecision |
| Muscle quantity / lean mass | Not serious | Not serious | Serious | Serious | Undetected; borderline signal | Low | Downgraded for serious class-level indirectness and serious imprecision |
| CSA / direct morphology | Not serious | Not serious | Serious | Very serious | Undetected; not assessable | Very low | Downgraded for serious class-level indirectness and very serious imprecision |

**Table Notes:** Certainty of evidence was assessed across five GRADE domains: risk of bias, inconsistency, indirectness, imprecision, and publication bias. Because all included studies were randomized controlled trials, the initial certainty was rated as high and downgraded where appropriate. Publication-bias judgments were exploratory because fewer than ten study clusters were available for each primary outcome domain. Overall certainty was classified as high, moderate, low, or very low. Because the synthesis estimated broad class-level effects across chemically distinct polyphenols, diverse populations and training protocols, and different measurement modalities, indirectness was judged serious for all three primary domains.

# 9 Supplementary Table S7

Table S7 Full exploratory subgroup analyses for primary outcome domains

| Outcome domain | Moderator | Subgroup | K | C | N | Hedges’ g [95% CI] | p | 95% PI | I²within | I²between | P-diff | CRVE P-diff (exploratory) |
| --- | --- | --- | --- | --- | --- | --- | --- | --- | --- | --- | --- | --- |
| Strength adaptations | Strength type | Dynamic RM strength | 14 | 5 | 141 | 0.32 [−0.10, 0.73] | 0.139 | −0.58 to 1.21 | 0.0% | 49.4% | 0.572 | 0.037 |
|  |  | Isometric/dynamometer strength | 4 | 3 | 84 | 0.04 [−0.35, 0.43] | 0.833 | −0.35 to 0.43 | 0.0% | 0.0% |  |  |
|  |  | Isokinetic strength | 4 | 1 | 49 | 0.43 [0.03, 0.83] | Not reported | Not reported | 0.0% | 0.0% |  |  |
|  | Population age group | Middle-aged/older participants | 12 | 5 | 193 | 0.28 [−0.05, 0.60] | 0.094 | −0.34 to 0.89 | 0.0% | 36.3% | 0.821 | 0.843 |
|  |  | Younger/adult participants | 10 | 3 | 64 | 0.22 [−0.32, 0.76] | 0.418 | −0.74 to 1.19 | 0.0% | 45.5% |  |  |
|  | Polyphenol class | Catechin/flavanol-rich polyphenols | 12 | 4 | 93 | 0.37 [−0.12, 0.86] | 0.138 | −0.60 to 1.35 | 0.1% | 49.6% | 0.460 | 0.491 |
|  |  | Other polyphenols | 10 | 4 | 164 | 0.14 [−0.13, 0.41] | 0.301 | −0.24 to 0.53 | 0.2% | 14.2% |  |  |
|  | Intervention duration | ≤8 weeks | 13 | 5 | 119 | 0.33 [−0.08, 0.74] | 0.110 | −0.51 to 1.18 | 0.1% | 43.1% | 0.530 | 0.528 |
|  |  | >8 weeks | 9 | 3 | 138 | 0.14 [−0.17, 0.46] | 0.365 | −0.32 to 0.61 | 0.2% | 21.1% |  |  |
| Muscle quantity / lean mass | Population age group | Middle-aged/older participants | 10 | 6 | 226 | 0.06 [−0.15, 0.28] | 0.575 | −0.15 to 0.28 | 0.0% | 0.0% | 0.092 | 0.338 |
|  |  | Younger/adult participants | 3 | 3 | 54 | 0.58 [−0.27, 1.43] | 0.180 | −0.82 to 1.97 | 28.4% | 28.4% |  |  |
|  | Polyphenol class | Catechin/flavanol-rich polyphenols | 4 | 4 | 83 | 0.59 [0.04, 1.14] | 0.035 | −0.25 to 1.43 | 16.6% | 16.6% | 0.023 | 0.086 |
|  |  | Other polyphenols | 9 | 5 | 197 | 0.01 [−0.21, 0.23] | 0.921 | −0.21 to 0.23 | 0.0% | 0.0% |  |  |
|  | Intervention duration | ≤8 weeks | 4 | 4 | 90 | 0.32 [−0.14, 0.78] | 0.170 | −0.51 to 1.15 | 23.5% | 23.5% | 0.299 | 0.348 |
|  |  | >8 weeks | 9 | 5 | 190 | 0.07 [−0.16, 0.30] | 0.544 | −0.16 to 0.30 | 0.0% | 0.0% |  |  |

**Table Notes:** K denotes effect sizes; C, study clusters; N, unique participants within each subgroup, participants contributing multiple effect sizes within the same subgroup were counted once; PI, prediction interval; P-diff, omnibus three-level moderator test; CRVE P-diff, CR2-corrected cluster-robust moderator test. All analyses were descriptive and hypothesis-generating. The isokinetic subgroup contained one cluster; its P value and PI were not reported. For strength type, the conventional moderator test (P = 0.572) and CRVE test (P = 0.037) were discordant; the latter used only eight clusters, approximately 3 denominator degrees of freedom, and a one-cluster subgroup, and was therefore not interpreted as evidence of effect modification. The muscle-quantity polyphenol-class contrast was not retained under CRVE (P = 0.086).

# 10 Supplementary Table S8

Table S8 Robustness analyses for within-study sampling-error dependence and study-cluster aggregation

| Outcome domain | Analysis | K/C/N | Hedges’ g [95% CI] | P value | df |
| --- | --- | --- | --- | --- | --- |
| Strength adaptations | Primary three-level REML: V = diag(vᵢ) | 22/8/257 | 0.25 [−0.01, 0.51] | 0.060 | — |
|  | CRVE–CR2 | 22/8/257 | 0.25 [−0.07, 0.57] | 0.105 | 6.61 |
|  | Block-diagonal V, ρ = 0.30 | 22/8/257 | 0.24 [−0.02, 0.49] | 0.075 | — |
|  | Block-diagonal V, ρ = 0.60 | 22/8/257 | 0.22 [−0.03, 0.47] | 0.090 | — |
|  | Block-diagonal V, ρ = 0.90 | 22/8/257 | 0.22 [−0.07, 0.50] | 0.141 | — |
|  | One effect per cluster, ρ = 0.30; REML–KH | 8/8/257 | 0.24 [−0.07, 0.54] | 0.113 | 7.00 |
|  | One effect per cluster, ρ = 0.60; REML–KH | 8/8/257 | 0.22 [−0.08, 0.51] | 0.129 | 7.00 |
|  | One effect per cluster, ρ = 0.90; REML–KH | 8/8/257 | 0.19 [−0.09, 0.46] | 0.151 | 7.00 |
| Muscle quantity/lean mass | Primary three-level REML: V = diag(vᵢ) | 13/9/280 | 0.13 [−0.07, 0.33] | 0.211 | — |
|  | CRVE–CR2 | 13/9/280 | 0.13 [−0.10, 0.35] | 0.207 | 5.49 |
|  | Block-diagonal V, ρ = 0.30 | 13/9/280 | 0.15 [−0.07, 0.37] | 0.175 | — |
|  | Block-diagonal V, ρ = 0.60 | 13/9/280 | 0.17 [−0.06, 0.41] | 0.151 | — |
|  | Block-diagonal V, ρ = 0.90 | 13/9/280 | 0.19 [−0.06, 0.43] | 0.137 | — |
|  | One effect per cluster, ρ = 0.30; REML–KH | 9/9/280 | 0.15 [−0.13, 0.44] | 0.253 | 8.00 |
|  | One effect per cluster, ρ = 0.60; REML–KH | 9/9/280 | 0.17 [−0.13, 0.47] | 0.222 | 8.00 |
|  | One effect per cluster, ρ = 0.90; REML–KH | 9/9/280 | 0.19 [−0.12, 0.50] | 0.204 | 8.00 |
| CSA/direct morphology | Primary three-level REML: V = diag(vᵢ) | 6/3/115 | 0.05 [−0.21, 0.30] | 0.718 | — |
|  | CRVE–CR2 | 6/3/115 | 0.05 [−0.33, 0.43] | 0.542 | 1.46 |
|  | Block-diagonal V, ρ = 0.30 | 6/3/115 | 0.05 [−0.25, 0.35] | 0.739 | — |
|  | Block-diagonal V, ρ = 0.60 | 6/3/115 | 0.05 [−0.28, 0.39] | 0.761 | — |
|  | Block-diagonal V, ρ = 0.90 | 6/3/115 | 0.05 [−0.31, 0.42] | 0.781 | — |
|  | One effect per cluster, ρ = 0.30; REML–KH | 3/3/115 | 0.05 [−0.17, 0.27] | 0.420 | 2.00 |
|  | One effect per cluster, ρ = 0.60; REML–KH | 3/3/115 | 0.05 [−0.17, 0.27] | 0.411 | 2.00 |
|  | One effect per cluster, ρ = 0.90; REML–KH | 3/3/115 | 0.05 [−0.17, 0.27] | 0.411 | 2.00 |

**Table Note:** K, effect sizes; C, study clusters; N, unique participants; ρ, assumed within-cluster sampling-error correlation; CRVE, cluster-robust variance estimation; KH, Knapp–Hartung; REML, restricted maximum likelihood. The primary model used V = diag(vᵢ). CRVE required no prespecified ρ. For CRVE, df denotes Satterthwaite degrees of freedom; for REML–KH, df = C − 1. CSA/direct morphology results were interpreted descriptively. All P values are two-sided.

Supplementary methods. A common ρ was applied to all retained effect pairs within a participant-source cluster, regardless of outcome, site, test, or measurement modality; between-cluster covariance was zero. This pragmatic assumption did not imply equal correlation. Shared-control arms were combined before effect-size calculation. All nine matrices were positive definite and required no correction. Aggregation was domain-specific and used no clinical hierarchy: ȳⱼ = (1ᵀVⱼ⁻¹yⱼ)/(1ᵀVⱼ⁻¹1) and Var(ȳⱼ) = (1ᵀVⱼ⁻¹1)⁻¹. The midpoint ρ = 0.60 was selected before the aggregated results were examined (not prespecified in PROSPERO), with repetitions at ρ = 0.30 and 0.90. Functions were base R outer(), solve(), and eigen(), metafor::rma.mv()/rma.uni(), and clubSandwich::coef_test().

# 11 Supplementary Table S9

Table S9 Sensitivity analyses across assumed pre–post correlations used to reconstruct missing change-score standard deviations

| Outcome domain | Kimp | Assumed r | K/C/N | Three-level Hedges’ g [95% CI] | P value | CRVE 95% CI | CRVE P value | df |
| --- | --- | --- | --- | --- | --- | --- | --- | --- |
| Strength adaptations | 12 | 0.30 | 22/8/257 | 0.23 [−0.01, 0.47] | 0.065 | [−0.07, 0.53] | 0.110 | 6.51 |
|  | 12 | 0.50 | 22/8/257 | 0.25 [−0.01, 0.51] | 0.060 | [−0.07, 0.57] | 0.105 | 6.61 |
|  | 12 | 0.70 | 22/8/257 | 0.29 [−0.01, 0.58] | 0.057 | [−0.07, 0.65] | 0.101 | 6.74 |
| Muscle quantity/lean mass | 8 | 0.30 | 13/9/280 | 0.12 [−0.08, 0.32] | 0.240 | [−0.10, 0.34] | 0.219 | 5.50 |
|  | 8 | 0.50 | 13/9/280 | 0.13 [−0.07, 0.33] | 0.211 | [−0.10, 0.35] | 0.207 | 5.49 |
|  | 8 | 0.70 | 13/9/280 | 0.14 [−0.06, 0.34] | 0.168 | [−0.10, 0.38] | 0.191 | 5.47 |
| CSA/direct morphology | 4 | 0.30 | 6/3/115 | 0.05 [−0.21, 0.30] | 0.714 | [−0.33, 0.42] | 0.534 | 1.46 |
|  | 4 | 0.50 | 6/3/115 | 0.05 [−0.21, 0.30] | 0.718 | [−0.33, 0.43] | 0.542 | 1.46 |
|  | 4 | 0.70 | 6/3/115 | 0.05 [−0.21, 0.30] | 0.726 | [−0.34, 0.43] | 0.558 | 1.46 |

**Table Note:** Kimp, effect sizes for which the change-score SD was reconstructed; r, assumed pre–post correlation. Only missing change-score SDs were recalculated; author-reported values were retained. The primary analysis used r = 0.50. CRVE point estimates equaled the corresponding three-level estimates; df denotes Satterthwaite degrees of freedom. CSA/direct morphology analyses were descriptive. All P values are two-sided.

# 12 Supplementary Table S10

Table S10 Alternative controlled pretest–posttest estimates standardized by the pooled baseline standard deviation

| Outcome domain | Assumed r | K/C/N | Change-score estimator, g [95% CI]; P | Pooled baseline-SD estimator, d_ppc2 [95% CI]; P | d_ppc2 with CRVE [95% CI]; P (df) |
| --- | --- | --- | --- | --- | --- |
| Strength adaptations | 0.30 | 14/5/150 | 0.26 [−0.07, 0.59]; 0.127 | 0.17 [−0.10, 0.45]; 0.221 | 0.17 [−0.25, 0.59]; 0.307 (3.52) |
|  | 0.50 | 14/5/150 | 0.27 [−0.08, 0.62]; 0.127 | 0.17 [−0.09, 0.43]; 0.205 | 0.17 [−0.23, 0.56]; 0.289 (3.63) |
|  | 0.70 | 14/5/150 | 0.30 [−0.09, 0.68]; 0.130 | 0.17 [−0.08, 0.41]; 0.185 | 0.17 [−0.20, 0.53]; 0.266 (3.78) |
| Muscle quantity/lean mass | 0.30 | 13/9/280 | 0.12 [−0.08, 0.32]; 0.240 | 0.09 [−0.15, 0.32]; 0.475 | 0.09 [−0.05, 0.22]; 0.173 (5.57) |
|  | 0.50 | 13/9/280 | 0.13 [−0.07, 0.33]; 0.211 | 0.08 [−0.11, 0.28]; 0.407 | 0.08 [−0.05, 0.22]; 0.170 (5.56) |
|  | 0.70 | 13/9/280 | 0.14 [−0.06, 0.34]; 0.168 | 0.08 [−0.07, 0.24]; 0.304 | 0.08 [−0.05, 0.21]; 0.165 (5.52) |
| CSA/direct morphology | 0.30 | 6/3/115 | 0.05 [−0.21, 0.30]; 0.714 | 0.01 [−0.30, 0.31]; 0.959 | 0.01 [−0.08, 0.10]; 0.649 (1.46) |
|  | 0.50 | 6/3/115 | 0.05 [−0.21, 0.30]; 0.718 | 0.01 [−0.25, 0.26]; 0.952 | 0.01 [−0.08, 0.10]; 0.649 (1.46) |
|  | 0.70 | 6/3/115 | 0.05 [−0.21, 0.30]; 0.726 | 0.01 [−0.19, 0.21]; 0.938 | 0.01 [−0.08, 0.10]; 0.649 (1.46) |

**Table Note:** d_ppc2 denotes Morris’s controlled pretest–posttest effect-size estimator standardized by the pooled baseline SD. Analyses were restricted to outcomes with sufficient baseline data; consequently, the strength analysis included 14 of 22 primary effect sizes. Both estimators were applied to the same outcome subset. The assumed r contributed to sampling-variance estimation. CRVE used CR2 correction, and df denotes Satterthwaite degrees of freedom. CSA/direct morphology analyses were descriptive. All P values are two-sided.

# 13 Supplementary Table S11

Table S11 Reports not retrieved and reasons for non-retrieval

| No. | Report | Source / DOI | Available record | Reason for non-retrieval |
| --- | --- | --- | --- | --- |
| 1 | Polyphenol Supplementation Does not Change the Post-Resistance Exercise Circulating and Intramuscular TNF-α or TNFR1 Response on Monocyte Subsets | FASEB Journal; 10.1096/fasebj.31.1_supplement.1019.2 | Conference abstract | No corresponding full-length report identified despite renewed searches. |
| 2 | Novel Form of Curcumin Attenuates Performance Decrements Following Muscle Damaging Exercise | FASEB Journal; 10.1096/fasebj.31.1_supplement.lb415 | Conference abstract | No corresponding full-length report could be retrieved. |
| 3 | Effects of Blackcurrant Supplementation on Exercise Performance in Resistance-Trained Athletes | Physiology; 10.1152/physiol.2025.40.S1.1864 | Conference abstract | Only the abstract record was available; no full-length report could be retrieved. |
| 4 | Blackcurrant Supplementation Enhances Cognitive Performance in Resistance-Trained Individuals | Physiology; 10.1152/physiol.2025.40.S1.1868 | Conference abstract | Only the abstract record was available; no full-length report could be retrieved. |
| 5 | Polyphenol Supplementation Alters Resting Metabolomic Profiles After Resistance Training in Older Adults | BMJ Open Sport & Exercise Medicine; 10.1136/bmjsem-2026-sportskongres.22 | Conference abstract | No corresponding full-length report identified despite renewed searches. |
| 6 | The Efficacy and Safety of Curcumin Combined with Resistance Exercise Program in Filipinos with Probable Sarcopenia | Journal of the Neurological Sciences; 10.1016/j.jns.2025.123975 | Meeting supplement abstract | No separate full-length report could be retrieved. |
| 7 | Effects of Combined Intervention of Quadriceps Exercise and Green Tea Catechins on Physical Function in Medial Knee Osteoarthritis | Osteoarthritis and Cartilage; 10.1016/j.joca.2017.02.683 | Conference abstract | No accessible full-length report could be retrieved through the searched sources. |

**Table Note:** All seven records were rechecked using publisher websites, bibliographic databases, and institutional library resources; however, no corresponding full-length reports were identified. Because only conference or meeting-supplement abstracts were available, final eligibility and extractable between-group effects could not be established. Their non-inclusion may have introduced availability bia

# 14 Supplementary Table S12

Table S12 Mapping of source reports, participant samples, study clusters, and analytical roles

| Cluster ID | Source report / trial component | Parent trial / participant sample | Strict-primary domain(s) | Additional analytical role |
| --- | --- | --- | --- | --- |
| C1 | Flensted-Jensen et al. 2025 | Single independent sample (n = 41) | Strength adaptations; muscle quantity / lean mass; CSA / direct morphology | Mixed RT + HIIT sensitivity |
| C2 | Nishikawa et al. 2025 | Single independent sample (n = 26) | Strength adaptations; muscle quantity / lean mass; CSA / direct morphology | — |
| C3 | Juesas et al. 2025 | Same parent trial and sample as Saez-Berlanga et al. (n = 49) | Muscle quantity / lean mass | Shared participant source |
| C3 | Saez-Berlanga et al. 2025 | Same parent trial and sample as Juesas et al. (n = 49) | Strength adaptations | Shared participant source |
| C4 | Shigeta et al. 2023, Trial 1 | Independent 8-week trial (n = 17) | Strength adaptations; muscle quantity / lean mass | — |
| C5 | Shigeta et al. 2023, Trial 2 | Independent 12-week trial (n = 19) | Muscle quantity / lean mass | Figure-estimated-data sensitivity |
| C6 | Otsuka et al. 2022 | Multi-arm trial with a shared placebo group (n = 48) | Strength adaptations; muscle quantity / lean mass; CSA / direct morphology | Active arms combined |
| C7 | Mafi et al. 2018 | Single independent sample (n = 29) | Strength adaptations; muscle quantity / lean mass | — |
| C8 | Beyer et al. 2017 | Single independent sample (n = 29) | Strength adaptations | — |
| C9 | Cardoso et al. 2013 | Single independent sample (n = 18) | Strength adaptations; muscle quantity / lean mass | Randomization-reporting sensitivity |
| C10 | Orsatti et al. 2010 | Single independent sample (n = 33) | Muscle quantity / lean mass | — |
| C11 | Tokuda and Mori 2021 | Single independent sample (n = 51) | None | EAA-matched add-on analyses only |

**Table note:** A *report* denotes a publication, a *trial* denotes an independently randomized experiment, and a *study cluster* denotes an independent participant source. Reports sharing participants were assigned the same cluster, whereas independent trials within one report were assigned separate clusters. Thus, Juesas et al. and Saez-Berlanga et al. formed C3, whereas the two Shigeta trials formed C4 and C5. All clusters contributing to a strict-primary domain were included in the corresponding complete leave-one-study-cluster-out analysis. C11 was excluded from strict-primary models because both groups received EAA. The abstract, primary results, GRADE assessments, and conclusions refer to the strict-primary models.

# 15 Supplementary Table S13

Table S13 Adverse-event reporting in the included reports

| Report | Safety information reported | Classification |
| --- | --- | --- |
| Flensted-Jensen et al. (2025) | No extractable trial-specific safety information | Not reported |
| Nishikawa et al. (2025) | No extractable trial-specific safety information | Not reported |
| Juesas et al. (2025) | No injuries or adverse effects attributed to the study procedures were reported | Explicit statement; monitoring method unclear |
| Saez-Berlanga et al. (2025) | No dropout, injury, or adverse response was attributed to the intervention | Explicit statement; monitoring method unclear |
| Otsuka et al. (2022) | Safety was actively assessed. No QG-related side effects or severe adverse events were observed; event incidence did not differ significantly among groups | Actively assessed |
| Tokuda and Mori (2021) | No extractable trial-specific safety information; reported withdrawals were due to consent withdrawal | Not reported |
| Shigeta et al. (2023; two trials) | No extractable trial-specific safety information | Not reported |
| Beyer et al. (2017) | No withdrawal or serious long-term adverse event was attributed to either treatment | Explicit statement; monitoring method unclear |
| Mafi et al. (2018) | No extractable trial-specific safety information | Not reported |
| Cardoso et al. (2013) | No extractable trial-specific safety information | Not reported |
| Orsatti et al. (2010) | Adverse experiences occurred in 20.0% of the soy group and 11.1% of the placebo group, most commonly gastrointestinal; no serious event was attributed to isoflavones | Adverse experiences reported |

**Table note:** “Not reported” indicates that no extractable trial-specific safety information was identified; it does not indicate that no adverse events occurred. Reports containing an explicit safety statement did not necessarily describe systematic adverse-event monitoring. Safety outcomes were not quantitatively synthesized, and no conclusion regarding intervention safety was drawn.

# 16 Supplementary Table S14

Table S14 Study-level reporting of adherence, dietary assessment, co-interventions, and training progression

| Trial/cluster | Supplement adherence | Training adherence and progression | Dropout/withdrawal | Diet and bioactive exposure | Co-intervention |
| --- | --- | --- | --- | --- | --- |
| Flensted-Jensen 2025 | Checklist; 98 ± 2%; none <90% | Attendance NR; load progressed from 12RM to 10RM, 8RM, and 6RM | NR | Usual diet; habitual polyphenol intake NR | Limited HIIT |
| Juesas/Saez-Berlanga 2025 | Self-report; 89.39% | 87.23–89.58% of sessions completed; supervised progressive elastic-band training | No injury-related withdrawal reported | Three-day dietary records; energy and macronutrient intake assessed; habitual polyphenol intake NR | None reported |
| Nishikawa 2025 | NR | Attendance NR; intensity standardized using real-time force feedback | NR | Diet and habitual polyphenol intake NR | None reported |
| Shigeta 2023, Trial 1 | NR | Attendance NR; machine loads adjusted around individual 10RM | NR | Diet and habitual polyphenol intake NR; placebo caffeine-matched | Caffeine-matched placebo |
| Shigeta 2023, Trial 2 | NR | Attendance NR; machine loads adjusted around individual 10RM | NR | Diet and habitual polyphenol intake NR; placebo caffeine-matched | Caffeine-matched placebo |
| Otsuka 2022 | Diary and capsule count; 99.5–99.9% | 95.6–97.0% attendance; progression monitored by instructors | Reported in the trial flow diagram | Background diet and habitual polyphenol intake NR | None reported |
| Mafi 2018 | NR | Attendance NR; 1RM reassessed every 2 weeks | NR | Three-day dietary records collected during the first and final weeks; energy and protein intake assessed | None reported |
| Beyer 2017 | Returned supplement containers; compliance >80% required | Attendance criterion applied; load increased after completion of the prescribed repetitions | Eighteen participants excluded for testing or compliance-related reasons | Dietary recalls; energy and macronutrient intake assessed | Polyphenol blend contained caffeine |
| Cardoso 2013 | NR | Attendance NR; progressive machine-based resistance training | NR | Hypocaloric dietary run-in; dietary records; coffee, black tea, and mate tea controlled | Hypocaloric dietary run-in |
| Orsatti 2010 | Unused capsules returned | Attendance recorded; gradual progression from 60% to 80% 1RM | Withdrawals and poor compliance reported | Usual diet assessed; habitual polyphenol intake NR | None reported |
| Tokuda and Mori 2021 | Post-session intake supervised or recorded; exact percentage NR | Attendance NR; load progressed from approximately 50% to 70% 1RM | Consent withdrawals reported | Nutritionist-supervised dietary assessment; five-day weighed records; protein intake maintained at ≥1.2 g/kg/day | Both groups received essential amino acids |

**Table note:** NR indicates that the information was not reported or was not extractable from the available report; it does not indicate that the procedure was not implemented. Numerical or qualitative information is presented only when explicitly reported in the primary study. RM, repetition maximum; HIIT, high-intensity interval training; EAA, essential amino acids.

# 17 Supplementary Figure S1

Figure S1 Classic three-level forest plot for strength adaptations

# 18 Supplementary Figure S2

Figure S2 Classic three-level forest plot for muscle quantity / lean mass outcomes

# 19 Supplementary Figure S3

Figure S3 Classic three-level forest plot for CSA / direct morphology outcomes

# 20 Supplementary Figure S4

Figure S4 Forest plot and sensitivity analyses for fat mass

# 21 Supplementary Figure S5

Figure S5 Funnel plot for fat mass / body composition

# 22 Supplementary Figure S6

Figure S6 Subgroup analysis of effect sizes (Hedges' g) for strength adaptations and muscle quantity

# 23 Supplementary Figure S7

Figure S7 CRVE-CR2 robustness of the primary three-level models

# 24 Supplementary Figure S8

Figure S8 Sensitivity to assumed within-cluster sampling-error correlations and study-cluster aggregation

# 25 Supplementary Figure S9

Figure S9 Sensitivity to assumed pre–post correlations used to reconstruct change-score standard deviations

# 26 Supplementary Figure S10

Figure S10 Alternative pooled-baseline-SD standardized controlled pretest–posttest estimator

# 27 Supplementary Figure S11

Figure S11 Complete leave-one-study-cluster-out analyses
